# Correction: Improved residual fat malabsorption and growth in children with cystic fibrosis treated with a novel oral structured lipid supplement: A randomized controlled trial

**DOI:** 10.1371/journal.pone.0239642

**Published:** 2020-09-17

**Authors:** Virginia A. Stallings, Alyssa M. Tindall, Maria R. Mascarenhas, Asim Maqbool, Joan I. Schall

The Competing Interests statement is incorrect. The correct Competing Interests statement is as follows: LXS (now known as Encala™) was a product of BioMolecular Products, Inc and was used for the clinical trial. Envara Health was founded in 2018 to develop therapeutic nutrition products related to the licensed LXS (now Encala™) technology. After study completion, VAS co-founded Envara Health and serves as a consultant.
