# Supplementary material for: Improved residual fat malabsorption and growth in children with cystic fibrosis treated with a novel oral structured lipid supplement: A randomized controlled trial
Source: PLoS One. 2020 May 8;15(5):e0232685. doi: 10.1371/journal.pone.0232685 (PMC7209323; doi:10.1371/journal.pone.0232685)
Supplement: S1 File — (PDF) [file pone.0232685.s001.pdf]

IRB PROTOCOL SUMMARY

LYM-X-SORB™, an Organized Lipid Matrix: Fatty Acids and Choline in CF

Note: Additions in bold italics indicate changes from original grant

Title: "LYM-X-SORB™, an Organized Lipid Matrix: Fatty Acids and Choline in CF"
IRB#: 2005-11-4611stallingsv
CTRC#: 2026
Study Key Name: StallingsV_05-004611 (Lym-X-Sorb)

Principal Investigator(s): Virginia A Stallings, MD

3535 Market St., Rm. 1558
Philadelphia, PA 19104
Tel: 215-590-1664
Fax: 215-590-0604
email: stallingsv@email.chop.edu

Co-Investigators: Maria Mascarenhas, MBBS; Asim Maqbool, MD; Joan Schall, PhD
Telephone: 215-590-1664 (Stallings)

Study Coordinator(s): Norma Latham, MHS

Study Research Technician: Thananya Wooden, BS

Sponsor: National Institutes of Health (NIDDK) **Location: Bethesda, MD**

SBIR Phase II grant: Small business site, Principal Investigator (Manufacturer of LYM-X-SORB™)
Walter Shaw, PhD., President
Avanti Polar Lipids, Inc.
700 Industrial Park Drive
Alabaster, AL 35007

OVERVIEW OF STUDY DESIGN

1. ABSTRACT

Individuals with cystic fibrosis (CF) and pancreatic insufficiency (PI) are prone to fat malabsorption, putting them at risk for caloric, essential fatty acid, and choline deficiency, which, in turn, may lead to growth failure and a poorer clinical course. Many subjects with CF have essential fatty acid deficiency, characterized by decreased levels of linoleic acid and an increased triene/tetraene ratio, and an associated choline deficiency. As a key membrane phospholipid, choline is required for methyl metabolism, cholinergic neurotransmission, transmembrane signaling, lipid cholesterol transport and metabolism. Choline deficiency is associated with liver disease, apoptosis, steatosis, as well as brain and visual development abnormalities. In a previous randomized control trial, supplementation with LYM-X-SORB™, an organized lipid matrix containing lysophosphatidylcholine (LPC) monoglycerides, and triglycerides, has been shown to improve fatty acid status and vitamin E and retinol binding protein levels over a 12-month period and to improve both growth and pulmonary function status over 18 months in subjects with CF. We propose to conduct a randomized placebo-controlled double-blinded study to evaluate the effectiveness of the next generation LYM-X-SORB™ with improved palatability and mixing characteristics, on fatty acid and choline status of 112 children, ages 5.0 to 18.9 years, with CF and PI. We propose that essential fatty acid status (linoleic acid levels, triene/tetraene ratio) and choline status (phosphadytlcholine/ phosphatidytlethanolamine (PC/PE) ratio) will be normalized

after 12 mos of supplementation with LYM-X-SORB™ in subjects receiving LYM-X-SORB™ (n=56) compared to those receiving placebo (n=56). We will also explore whether LYM-X-SORB™ supplementation will improve fat soluble vitamin status, bile composition, incidence of fatty liver, inflammatory cytokines, resting energy expenditure and respiratory quotient over 12 mos and improve pulmonary function, growth status, body composition, bone health and overall health status over 18 mos. Subjects will be recruited from ten Cystic Fibrosis Centers and have four major protocol visits to CHOP (baseline, 3, 12, and 18 mos), and one visit at their home Center (6 mos). Our objective is to determine if LYM-X-SORB™ can be used as an acceptable, effective, supplement to correct the metabolic and physiological abnormalities associated with fat malabsorption in subjects with CF, the most commonly inherited genetic disease in Caucasians.

2. LAY ABSTRACT

Fat malabsorption is common in Individuals with cystic fibrosis (CF) and pancreatic insufficiency (PI). This places them at risk for caloric, essential fatty acid, and choline deficiency, which may, in turn, lead to growth failure and a poorer clinical course. Many subjects with CF have essential fatty acid deficiency, characterized by decreased levels of linoleic acid and an increased triene/tetraene ratio, and an associated choline deficiency. Choline deficiency is associated with liver disease as well as brain and visual development abnormalities. In a previous randomized control trial, supplementation with LYM-X-SORB™, a new type of dietary fat supplement made up of naturally occurring fats put together in a way that makes them easier to absorb, has been shown to improve fatty acid status and vitamin E and vitamin A status over a 12-month period and to improve both growth and pulmonary function status over 18 months in subjects with CF. We propose to conduct a randomized placebo-controlled double-blinded study to evaluate the effectiveness of the next generation LYM-X-SORB™ with better taste and mixing characteristics, on fatty acid and choline status of 112 children, ages 5.0 to 18.9 years, with CF and PI. We propose that essential fatty acid status (linoleic acid levels, triene/tetraene ratio) and choline status (phosphadytlcholine/ phosphatidytlethanolamine (PC/PE) ratio) will be normalized after 12 months of supplementation with LYM-X-SORB™ in subjects receiving LYM-X-SORB™ powder (n=56) compared to those receiving a placebo powder (n=56). We will also explore whether LYM-X-SORB™ supplementation will improve vitamin status (vitamins A, E, D and K), incidence of fatty liver, resting energy expenditure, and reduce inflammatory responses over 12 mos and improve pulmonary function, growth status, body composition, bone health and overall health status over 18 mos. Subjects will be recruited from nine Cystic Fibrosis Centers and have four major protocol visits to CHOP (baseline, 3, 12, and 18 mos), and one visit at their home Center (6 mos). Our objective is to determine if LYM-X-SORB™ can be used as an acceptable, effective, supplement to correct the metabolic and physiological abnormalities associated with fat malabsorption in subjects with CF, the most commonly inherited genetic disease in Caucasians.

3. TIME AND EVENTS SCHEDULE (Original Proposal)

Lymxsorb Schedule of Subject Visits (n = 78 – with five protocol visits)

	Year 1 (2006-7)												Year 2 (2007-8)												Year 3 (2008-9)											
	Jul	Aug	Sep	Oct	Nov	Dec	Jan	Feb	Mar	Apr	May	Jun	Jul	Aug	Sep	Oct	Nov	Dec	Jan	Feb	Mar	Apr	May	Jun	Jul	Aug	Sep	Oct	Nov	Dec	Jan	Feb	Mar	Apr	May	Jun
**Start-up, Training**																																				
**Baseline Admission (1)**					7	6	7	6	7	6	7	6	7	6	7	6																				
**3-month followup (1)**								7	6	7	6	7	6	7	6	7	6	7	6																	
**6-month followup (2)**											7	6	7	6	7	6	7	6	7	6	7	6														
**12-month Admission (1)**																	7	6	7	6	7	6	7	6	7	6	7	6								
**18-month Admission (3)**																							7	6	7	6	7	6	7	6	7	6	7	6		
**Data analysis**																																				
**Total**					7	6	13	13	13	19	20	19	20	19	20	19	20	19	20	12	14	12	14	12	14	12	14	12	7	6	7	6	7	6		

	Baseline	3mo	6mo	12mo	18mo	Grand Total
Year 1	52	33	13			98
Year 2	26	45	65	52	13	201
Year 3				26	65	91
Total	78	78	78	78	78	390

1 = CHOP overnight visit, GCRC
2 = Home center visit
3 = CHOP GCRC – day visit

ABBREVIATIONS

**CF**	Cystic Fibrosis
**CFTR**	Cystic fibrosis transmembrane regulator
**CHOP**	Children's Hospital of Philadelphia
**DRI**	Dietary Reference Intake
**DXA**	Dual energy x-ray absorptiometry
**EFA**	Essential fatty acid
**FA**	Fatty acid
**FEV_1**	Forced expiratory volume at one second, % predicted
**FM**	Fat mass, kg
**FFM**	Fat-free mass, kg
**GC/FID**	Gas chromatography with flame ionization detector
**GCRC**	General Clinical Research Center, CHOP
**HPLC**	High performance Liquid Chromatography
**HS-CRP**	High sensitivity C reactive protein
**LPC**	Lysophosphatidylcholine
**MG**	Monoglyceride
**MIP/MEP**	Maximum inspiratory pressure/ maximum expiratory pressure
**MRI/MRS**	Magnetic resonance imaging/ magnetic resonance spectroscopy
**MS/MS**	Triple quadrupole mass spectrometry
**PC**	Phosphatidylcholine
**PC/PE**	Phosphatidylcholine/phosphatidylethanolamine ratio
**PE**	Phosphatidylethanolamine
**PI**	Pancreatic insufficiency
**PLA2**	Phospholipase A2
**PQCT**	Peripheral quantitative CAT scan
**PUFA**	Polyunsaturated fatty acid
**RBC**	Red blood cell
**RBP**	Retinol binding protein
**REE**	Resting energy expenditure
**RQ**	Respiratory quotient
**SM**	Sphingomyelin

SPECIFIC AIMS and HYPOTHESES

Over the last 3 decades, the median age of survival of people with cystic fibrosis (CF) has dramatically increased from 14 years in 1969 to 33 in 2003 [1]. With this clinical success, CF is now both a pediatric and adult disease. Growth and nutritional status in people with CF are related to the severity of lung disease, pancreatic insufficiency (PI) and to nutrient intake and absorption. Considering national data [1], nutrition-related growth failure is still at an unacceptably high rate. In 2003, 15% of patients with CF were <5th percentile for height and 16% were <5th for weight. When the 10th percentile for weight is used as a more appropriate screen for patients with a nutritionally high-risk condition such as CF, then 26% of the patients are below the desirable level [2]. Except in end stage lung disease, most of this growth failure and poor nutritional status is the consequence of inadequately treated PI and fat malabsorption [1, 2].

Because individuals with CF and PI are prone to fat malabsorption, they are at risk for caloric, essential fatty acid (EFA) and choline deficiency, which in turn, leads to growth failure and a poorer clinical course. Many subjects with CF have EFA deficiency, characterized by decreased levels of linoleic acid and an increased triene/tetraene ratio [3], and an associated choline deficiency [4-6]. As a key membrane phospholipid, choline is required for methyl metabolism, cholinergic neurotransmission, transmembrane signaling, lipid cholesterol transport and metabolism. Choline deficiency is associated with liver disease, apoptosis, steatosis, as well as brain and visual development abnormalities. In a previous randomized control trial, supplementation with the 1st generation LYM-X-SORB™, an organized lipid matrix containing lysophosphatidylcholine (LPC) monoglycerides, and triglycerides, has been shown improve EFA status and vitamin E and retinal binding protein levels over a 12 month period and to improve both growth and pulmonary function over 18 months in subjects with CF [7]. The goal of this study is to evaluate the effectiveness of the next generation LYM-X-SORB™ with improved palatability and mixing characteristics, on the EFA and choline status in a sample of 112 children, ages 5.0 to 18.9 years, with CF and PI, using a randomized, double blind, placebo-controlled design.

Primary Aim: To evaluate the effectiveness of the next generation LYM-X-SORB™ with improved palatability and mixing characteristics, to improve the EFA and choline status for children, ages 5.0 to 18.9 years, with CF and PI using a randomized, double blind, placebo-controlled design.

H1: In subjects with CF and PI, EFA status will improve, as indicated by increased serum linoleic acid and decreased triene/tetraene ratio, after 12 months of supplementation with LYM-X-SORB™ compared to placebo.

H2: In subjects with CF and PI, choline status will improve, as indicated by an increased serum phosphatidylcholine (PC) to phosphatidylethanolamine (PE)ratio (PC/PE), after 12 months of supplementation with LYM-X-SORB™ compared to placebo.

Secondary Aims: To describe the long-term changes in metabolic, inflammatory, growth, nutritional, pulmonary, and bone health status with LYM-X-SORB™ supplementation compared to placebo in subjects with CF and PI.

H3: In subjects with CF and PI, those receiving 12 months of LYM-X-SORB™ supplementation will show improvements in EFA status (serum fatty acid profile), choline status (serum LPC, homocysteine, methionine, vitamins B_6, B_{12}, acyl carnitine, and RBC folate, fecal PC), fat soluble vitamin status, bile acid composition, incidence of fatty liver, inflammatory cytokines, resting energy expenditure and respiratory quotient compared to those receiving placebo.

H4: In subjects with CF and PI, those receiving 18 months of LYM-X-SORB™ supplementation will show improved growth, body composition, pulmonary status, and bone health status compared to those receiving placebo.

INTRODUCTION: BACKGROUND AND RATIONALE

CF is one of the most common chronic, multi-system inheritable diseases in the United States, affecting more than 30,000 individuals nationwide. Children with CF, in addition to pulmonary disease, suffer from gastrointestinal disease and the nutritional consequences of PI, which affects 93% of patients [2]. These consequences include malnutrition, fat malabsorption, EFA and choline deficiency and fat-soluble vitamin deficiency, and growth failure [8]. The treatment of fat malabsorption and its consequences remains a challenge for the CF care team. LYM-X-SORB™, which does not require pancreatic enzyme activity for absorption, will improve the nutritional status and clinical outcomes for people with CF and PI.

1. CF and Fatty Acid Status

Dietary fat malabsorption in CF is only partially corrected with pancreatic enzymes [9]. Fatty acid status is also not entirely corrected, and may lead to other metabolic derangements noted in otherwise well-nourished young patients with CF [8, 10]. Additionally, alterations in membrane fatty acid profiles were reported in both pancreatic sufficient and insufficient patients as compared to normal controls [3]. EFA deficiency is well described in the CF population [11, 12]. It is characterized by decreased levels of 9,12-octadecadienoic acid, common name linoleic acid (18:2n-6) a fatty acid not synthesized by humans, and an increase in the triene/tetraene ratio (20:3n9/20:4n6 ratio). 5,8,11-Eicosatrienoic acid (20:3n9), also know as mead acid, is the only polyunsaturated fatty acid of note produced *de novo* by animals (Figure 1).

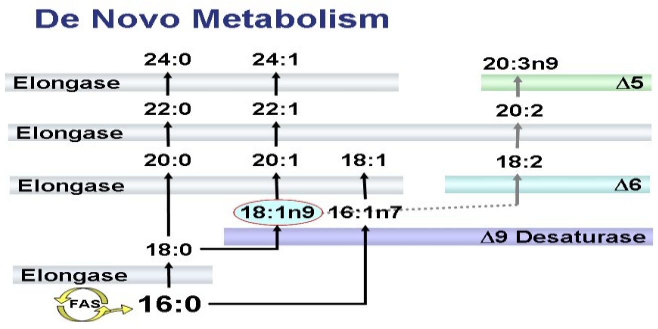

Figure 1.

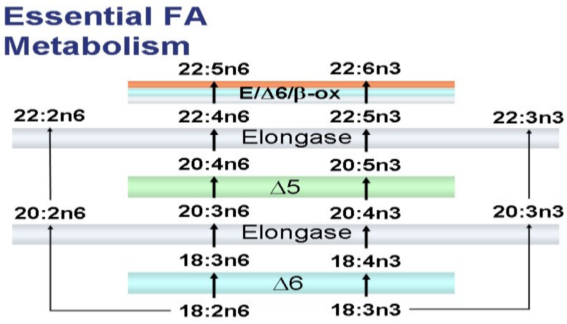

Figure 2.

Mead acid is rare in all lipid classes and accumulates under conditions of EFA deficiency. 5,8,11,14-Eicosatetraenoic acid (20:4n6), common name arachidonic acid, is derived from linoleic acid (18:2n6) (Figure 2) and is present in all lipid classes and particularly enriched in cholesterol esters, phosphatidylcholine, phosphatidylinositol and phosphatidylethanolamine. Biochemical evidence of EFA deficiency is common in CF, and precedes clinical signs [13]. EFA deficiency has been associated with impaired growth [14], hepatobiliary disease [15], increased vulnerability to lung infection [16], relationship to genotype [17] and the fluidity of plasma phospholipids [13]. EFA deficiency has been postulated to predispose to lung disease [16] and effect pulmonary function [18], derangements in fat-soluble vitamin status, such as vitamins A and E [19-21], with an increased inflammatory response [22]. Associations of EFA deficiency with growth status and energy balance have been demonstrated by trials of linoleic acid supplementation, omega 3 fatty acids in children with CF, effecting positive responses in body weight, growth, and EFA status [14, 23, 24].

2. CF and Choline Status

EFA deficiency may lead to an associated choline deficiency in subjects with CF. Choline and its phospholipids derivatives are fatty acids present in many foods, and are essential components of normal membrane structure and function, acetylcholine metabolism, cholinergic neurotransmission, transmembrane signaling, and lipid transport. Choline metabolism also intersects with the one-methyl and folate metabolic pathways. Choline derivatives play important roles in kidney and hepatobiliary function, central nervous system [25] and visual development, as well as spatial memory [26]. Endogenous de novo synthesis of choline occurs, prominently intra-hepatically via methylation of the precursor PE, with the methyl groups obtained from the one methyl pathway. The demand for choline varies throughout the life cycle, and observations in

animals and humans fed deficient diets detailed below have led the Institute of Medicine to classify choline as an essential nutrient in the recent review of the recommended dietary intakes [27].

Cells in culture die by apoptosis when deprived of choline, methionine, tryptophan or isoleucine [28]. Choline deficient diets in laboratory animals caused growth retardation, liver dysfunction (fatty infiltration, hepatocarcinoma) [29, 30], renal dysfunction, and hemorrhage [31]. Patients given total parenteral nutrition solutions lacking choline with sufficient folate and methionine developed fatty infiltration of the liver, which was reversible with choline supplementation [32] In low choline states, insufficient PC exists for VLDL production resulting in the accumulation of hepatic triglycerides. Choline, one methyl, folate, B_6 and B_{12} , and nucleotide metabolism are all linked [33]. The diet contains both choline and choline esters. Bioavailability of choline is based upon its ability to be enzymatically rendered into free choline and LPC, subsequently undergoing emulsification by bile acids, absorbed by enterocytes, re-esterified, and transported to the liver. Studies of intestinal tissue in individuals with CF have demonstrated diminished amounts of PLA2, the enzyme required to convert PC into LPC [34]. Stool specimens for phospholipids suggest that children with CF malabsorb lysophosphatidyl-choline and PC, compared to age matched healthy controls [35].

The predominant phospholipid in bile is PC, which originates from a hepatic pool, with a fraction contributed from the circulating lipoprotein pool, predominantly, from HDL. Biliary lipid secretion (phospholipids, cholesterol, plasma sterols, bile salts) functions in cholesterol homeostasis, and facilitates fat absorption from the intestinal lumen. Bile acid hydrophobicity is an important factor in this process, as well as in bile acid secretion rates. Individuals with CF have lower total bile acid output [36]. Abnormalities in biliary lipid composition in individuals with CF have been documented [37]. Malabsorption of bile acids in children with CF [38], defects in enterohepatic circulation [39] and defects in intraluminal metabolism of biliary PC to LPC may contribute to bile acid loss, and, thus, fat and PC malabsorption. Studies by Chen, Innis and colleagues have demonstrated that individuals with CF malabsorb dietary phospholipids including PC and LPC more than control subjects [5, 6]. Studies conducted in subjects with CF suggest increased turnover of some membrane phospholipids, as in platelets and fibroblasts [40]. Deficiency states of membrane phospholipids and of long-chain fatty acids have been demonstrated in children with CF and impact membrane fluidity function [13]. Plasma sterols may also be altered in people with cystic fibrosis, including abundant and less abundant sterols. Normative data in subjects with CF and normal controls examining PC and its precursor, as well as metabolites that intersect with one-methyl donor metabolism suggest choline deficiency in individuals with CF [4, 41]. A recent study by Chen et al [6] showed greater fecal loss of PC and LPC in subjects with CF compared to controls, and this loss was associated with altered plasma homocysteine, s-adenosylhomocysteine and methione, all of which intersect choline metabolism along the one-methyl metabolic pathway. In particular, in CF, fecal PC and LPC losses were positively correlated with plasma homocysteine and s-adenosylhomocysteine, and inversely correlated with plasma methionine.

3. CF and Liver

The involvement of the liver and biliary tract is increasingly recognized as a major CF manifestation [42]. Hepatic CFTR gene expression results in CFTR protein presence in the apical domains of the intra- and extra-hepatic bile ducts as well as in the gallbladder, and is most likely involved in chloride and water secretion into the canalicular system. Defects in this transmembrane protein function may effect the composition, fluidity, and flow of bile. The spectrum of hepatobiliary disease in individuals with CF is quite variable. Hepatic inflammatory reaction, fibrosis, and eventually cirrhosis are seen in association with these lesions over time [43]. Bile duct plugging is thought to be the cause of neonatal cholestasis seen in neonates with CF, along with meconium ileus and parenteral nutrition associated injury. Lastly, hepatic steatosis, seen in between 20-73% of individuals with CF may be related to malnutrition, EFA deficiency, choline deficiency or some as yet undetermined dietary or environmental factor [17, 44]. Choline deficiency is associated with non-alcoholic steatohepatitis (NASH), and replacement with either choline or its oxidative metabolite betaine, have been demonstrated to improve, or reverse the hepatosteatosis [32, 45]. The significant variability in incidence, type, onset and progression and severity of liver disease despite the same genotype, suggests additional modifiers. EFA deficiencies, on the other hand, have been shown to vary according to genotype, which may suggest a link between the basic genetic defect and abnormal fatty acid metabolism, in both pancreatic sufficient and insufficient patients [17]. Correction of EFA deficiency in children has resulted in improved growth and respiratory function [23], and can be affected by supplementation as seen in both animal models and studies performed in

children [24]. Similarly, replacement of choline may effect physiological changes in membrane function, platelet activity, hepatobiliary function, respiratory, nutritional, and growth status. We will use an MRI of the liver on a subset of subjects in this study to assess hepatic steatosis and MRS to quantify hepatic triglyceride content [46-48] at baseline and then the follow-up MRI/MRS at 12 months will document changes with LYM-X-SORB™ or placebo supplementation after 12 months. Non-invasive 1H MRS has proven to be an important tool for muscle lipid metabolism studies [49]. Its ability in detecting intra- and extra-myocellular lipids and other metabolites such as creatine and choline makes it a powerful tool for our study to follow changes in lipid and choline (trimethylamine) levels simultaneously.

4. CF, Inflammation and EFA

Humans are unable to synthesize fatty acids with the first double bond in the n-3 position, and, therefore, they are essential dietary nutrients. Desaturases and elongases then convert them to longer chain polyunsaturated fatty acids (PUFA). Cell membranes require unsaturated fatty acids to maintain structure, fluidity, and function. Eicosanoids, long chain fatty acids whose precursors are of chain lengths of 20 or more carbons, are of both the n-3 and n-6 type. Eicosanoids and their relative ratios of n-3:n-6 types influence immune activity by modulating the metabolites of cyclooxygenase pathways, including leukotrienes, prostaglandins, thromboxanes, and arachidonic acid derivatives. Arachidonic acid (AA; n-6) is pro-inflammatory, whereas eicosapentaenoic and docosahexanoic acids (EPA, DHA; n-3) are anti-inflammatory and down regulate the immune response. EFA deficiency impairs cell-mediated immune responses. Additional eicosanoid immune function is postulated via cytokines [50] and signal transduction pathways. These mechanisms have been studied in cardiovascular, inflammatory bowel, and pulmonary disease. EFA deficiency has been described in relation to genotype [17]; there is some evidence that the delta F508 CF gene mutation is associated with defects in EFA metabolism, with pathways affecting AA metabolism [51]. TNF-α levels have also been noted to be elevated in individuals with CF, and correlations to EFA deficiency have been documented [52]. Dietary supplementation trials with n-3 PUFA have been shown to decrease inflammatory cytokines and acute phase proteins in dyslipidemic subjects [53] as well as in children with CF, with studies in the latter demonstrating decreasing membrane AA levels, improved pulmonary function and decreased antibiotic use [54]. Serum leukotriene B4 (LTB4) levels, an inflammatory marker, were significantly reduced by n-3 PUFA compared to n-6 PUFA supplementation [55].

5. LYM-X-SORB and CF

LYM-X-SORB™ is an organized lipid matrix comprised of LPC monoglycerides, and fatty acids with defined mole ratios (1:4:2 to 1:2:4, respectively). LYM-X-SORB™ is composed of enzyme-modified PC, i.e. LPC, monoglycerides (MG) and fatty acids (FA), which are Generally Regarded As Safe (GRAS) listed by the FDA. The LYM-X-SORB™ components provide ample amounts of polyunsaturated fatty acids (PUFA), as they constitute 50% of the total fatty acids. The n6:n3 molar ratio of approximately 5:1 in LYM-X-SORB™ was maintained in this plasma mole ratio in subjects with CF [7]. In the present study the EFA will be normalized by supplementation of LYM-X-SORB™ as evidenced by serum linoleic acid levels and improved triene/tetraene ratio. The fatty acid composition of the serum PC will be determined.

LYM-X-SORB™ contains 20% LPC by weight. As noted earlier, the biliary secretion of 10 to 15 gm/day of PC and its eventual loss due to the absence of pancreatic phospholipase A_2 in PI requires patients with CF to synthesize both choline and PC *de novo*. To support this possibility, investigators [40] have shown that there was an increased turnover of PC in platelets of subjects with CF. Daily consumption of LYM-X-SORB™ provides approximately 5 gm of LPC and will replace half the daily loss of PC. Recently, Chen, Innis and colleagues [4, 6] have shown that changes in homocysteine, s-adenosylhomocysteine and methionine were associated with altered PC and PE molar ratios in CF. Therefore, supplementation of LYM-X-SORB™ will potentially result in changes in choline status and PC/PE ratio, and also homocysteine, s-ademosylhomocysteine and methionine, approaching levels in healthy subjects. LPC promotes the intestinal absorption of monoglycerides and fatty acids; whereas PC normally secreted in bile, inhibits their absorption [56, 57]. Gaskin et al [39, 58] indicated that 10 to15 gm/day of biliary secreted PC was not metabolized to LPC in subjects with CF and PI, thus, contributing to the fat malabsorption. LPC interacts with both MG and FA to form an organized lipid matrix [59]. LYM-X-SORB™ maintains its integrity in the milieu of the stomach and intestine and is quantitatively absorbed in both subjects with CF without enzyme supplement and healthy

subjects [60]. The results of orally consuming LYM-X-SORB™ in a double blind one-year study, with triglyceride as the control, have shown significant improvement in the clinical well-being of subjects with CF [7, 61]. Significant improvement was observed in terms of energy intake from diet, weight-for-age Z score, EFA status, vitamin E, RBP and, at the end of an additional 6-months observation period, significant improvement was also observed for height-for-age Z score and FEV_1.

The acute and long-term absorption of the fatty acid components of LYM-X-SORB™ has been significantly improved in subjects with CF [7]. However, the consequences of the absorption of the LPC component (choline) of LYM-X-SORB™ were not investigated. Assessment of stool choline plasma PC/PE ratio, one-methyl metabolites will document the effect of LYM-X-SORB™ supplementation on stool and plasma choline status [4, 6]. Maintaining an appropriate pool of PC *in situ* may be important for optimizing several physiological functions that are compromised in subjects with CF and that require PC (Figure 3).

<u>a. Cystic Fibrosis Transmembrane Regulator (CFTR):</u>
CF is characterized by elevated concentrations of electrolytes in the sweat [62]. CFTR is a conductance regulator as well as a chloride-channel [63]. CFTR can be functionally activated by LPC [64] and by treatment with phospholipase A_2 [65, 66], indicating that PC is an integral component of CFTR. Phospholipid chaperones may be important for CFTR trafficking [67]. Since patients with CF lose 10 to 15g of PC per day through biliary secretion, the inevitable reduction of the PC pool may affect the functionality of residual CFTR. Replenishing the PC pool by administering LYM-X-SORB™ may therefore improve the functionality of CFTR.

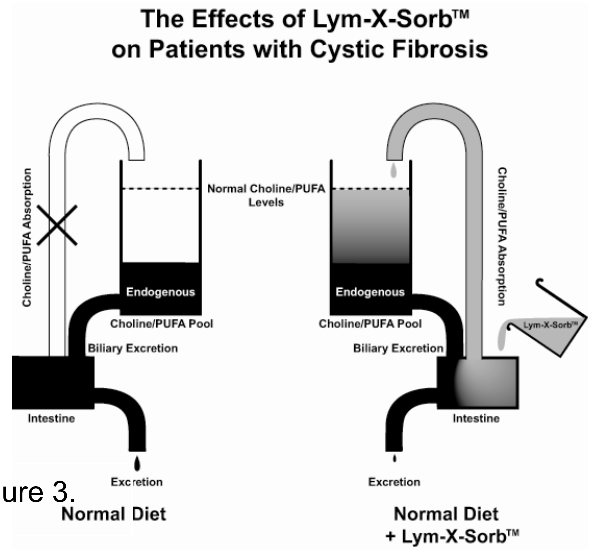

Figure 3.

<u>b. Biliary Bile Salts and PC</u>: Patients with CF are known to have a variety of bile acid abnormalities; increased fecal bile acid losses [38, 68-70], reduced bile acid pool size [71] and reduced duodenal bile acid concentration [37]. Durie and colleagues [36] have suggested that bile acid secretion is unrelated to fat malabsorption. Ninety-five percent of the phospholipids in bile are secreted as PC [72]. Nilsson, Scherstén and others showed that the enterohepatic circulation of bile acids was essential for the biliary secretion of PC [73, 74]. Others showed that the biliary excretion rate of PC was related to the bile acid excretion rate in a nonlinear way [75-77]. In addition, the fatty acid composition of the PC in bile varied with the type of bile salts [72]; there was a positive correlation between cholic and chenodeoxycholic acids and PC-linoleic acid and a negative correlation between deoxycholic acid and PC-linoleic acid. Conversely, negative correlations were found between cholic acid and PC-arachidonic acid, similarly between chenodeoxycholic acid and PC-arachidonic acid. There was a positive correlation between deoxycholic acid and PC-arachidonic acid. In healthy subjects, palmitic acid (41%) and linoleic acid (33%) were the major FA in biliary PC and arachidonic acid represented 6% [77].

The involvement of the liver and biliary tract is increasingly recognized as a major CF manifestation [42]. The pathogenesis of CF hepatobiliary disease is unknown and present treatment is unsatisfactory. There is evidence that PC increases bile secretion and prevents bile-acid induced cholestasis in rats [78], and that PC decreases fibrosis and bile duct proliferation in the MDR_3 knockout mouse model [79]. Based upon these observations, an LPC medical food may modify membrane composition, improve the CF biliary lipid secretion and thereby have a positive impact on liver disease. In patients with CF, the biliary secretion of bile salts is decreased [37, 38, 68-71], and the plasma 18:2n-6 is significantly lower than controls before treatment and increased to control levels after LYM-X-SORB™ consumption [7]. Therefore, normalization of plasma 18:2n-6 may correspond to an increased biliary PC enriched in 18:2n-6 with improved secretion of specific bile salts; e.g., cholic acid. The proposed research will focus on the status of both bile salts in serum and urine after daily consuming LYM-X-SORB™, containing 5 gm of LPC. In addition, MRI/MRS of the liver may also show improvement in fatty liver conditions over the 12 month study.

<u>c. Respiratory Quotient (RQ) and Resting Energy Expenditure (REE):</u> Mitochondria are self-replicating organelles whose primary function is the beta-oxidation of long chain fatty acids to yield high-energy

phosphates. Mitochondria membrane contains large quantities of PC. Mitochondrial dysfunction is an early indicator of apoptosis [80]. This is in agreement with earlier investigations showing twofold increases in the activities of individual mitochondrial enzymes with exercise training in humans [81, 82]. Interestingly, many functions associated with energy metabolism and the mitochondrial membranes are not operating properly in patients with CF [83]. Several groups have shown that aerobic fitness in patients with CF can be improved by exercise training [84, 85]. Also, higher levels of aerobic fitness are associated with lower mortality risk [86]. Healthy subjects with higher aerobic fitness also have reduced all-cause mortality [87]. Thus, the increased oral absorption of LPC in CF may enhance the synthesis of PC, *de novo*, for the increased production of mitochondria, its oxidation of fat and its production of high-energy phosphate for work and heat out-put. REE in patients with CF is higher than normal [88-90], and increases as lung function declines [91]. Substrate utilization at rest indicates a greater metabolism of carbohydrates in subjects with CF than in control subjects [88]. Mild exercise results in a greater percentage of carbohydrate utilization. There is also evidence that, as lung function deteriorates, there is a non-linear increase in metabolic rate [92]. During the LYM-X-SORB™ study, FEV_1 progressively improved [7]. Thus, during the improvement in lung capacity for work, improvement in FEV_1, should parallel the improvement (decrease) in RQ, indicating that the metabolism of fat is returning to normal. In summary, the consumption of LYM-X-SORB™ should provide sufficient absorption of LPC for the improved synthesis of PC *in situ* and the improved muscle mitochondrial oxidation of fat. The RQ provides a measure of carbohydrates and fat utilization during REE. After 12 months of supplementation, the RQ of the LYM-X-SORB™ group should decrease indicating normalization of fat metabolism compared to no change in the placebo group.

d. Pancreatic Function: Fat malabsorption is a salient feature of PI and CF. PC adsorption to dietary fats interferes with intraluminal fat digestion. Pancreatic phospholipase A2 (PLA2) hydrolyzes PC into LPC, removing it from other intraluminal fats, allowing for intraluminal fat digestion [58]. Furthermore, PLA2 requires both LPC and fatty acid in order to quantitatively hydrolyze PC [93, 94]. Individuals with CF lack PLA2, and pancreatic enzyme replacement only partially corrects this deficiency-associated fat malabsorption. If the pancreas has restored its capacity to produce a functional PLA2 enzyme with ingestion of LYM-X-SORB™ (by the delivery of LPC), then the secreted biliary PC in PI subjects may be processed to LPC and the normalization of fat absorption should be apparent. The resultant newly hydrolyzed LPC should be absorbed and incorporated into serum PC. Likewise, the degree of fat and choline malabsorption measured at baseline and 3 months by fecal fat quantification, should decrease. This improvement in malabsorption should be reflected in improved measurable membrane fatty acid and choline ester content, and effect physiological changes.

e. Growth, Nutritional and Pulmonary Status: As a group, children with CF and pancreatic insufficiency (PI) often have poor growth and nutritional status, delayed maturation and experience a decline in pulmonary function over time. Malnutrition and poor growth in CF results from chronic negative energy balance resulting from insufficient caloric intake coupled with higher energy requirements due to both increased energy loss from malabsorption and increased energy expenditure. In the previous double blind study of evaluating LYM-X-SORB™ and TG in control subjects and subjects with CF, additional parameters were improved in the LYM-X-SORB™ group [7]. In children, significant clinical improvement in dietary intake, EFA status, vitamin E [7], RBP, and weight-for-age Z score occurred and, at the end of an additional six months, significant improvements were observed for height-for-age Z score and lung function (FEV1 % predicted). During our proposed 18-month trial, growth and nutritional status parameters, sexual maturity status and pulmonary function will be monitored. Improved nutritional status and choline status may also result in improved voluntary muscle, pulmonary function and respiratory muscle strength [95, 96]. Therefore, respiratory and peripheral muscle function will be assessed by measuring maximum inspiratory and expiratory pressure [97, 98]. Since we have previously found that infection of the lungs is an important confounder for pulmonary function, we will obtain a sputum culture to determine presence/absence of microorganism colonization so that we can better assess improvement of pulmonary function as a long-term outcome of LYM-X-SORB™ supplementation. The previous LYM-X-SORB™ group noted that their stools were less malodorous, bulky and greasy and in our proposed study, steatorrhea and enzyme use will be monitored. There was also a trend in the LYM-X-SORB™ group for fewer intravenous antibiotic days, and so the clinical records will be

monitored. In toto, these parameters will provide information about the clinical well-being of the subjects with CF receiving either LYM-X-SORB™ or placebo.

f. Quality of Life: Cystic Fibrosis is a chronic and fatal disease and affects children and adolescents, not only physically, but psychologically, emotionally and socially, thereby affecting quality of life. It is well known that health-related quality of life (HRQOL) measures are an important outcome in health research and are a more meaningful way of describing health status. Using these measures, one can not only identify subgroups of children who are at risk for health problems, but the burden of disease can be determined and appropriate efforts at prevention and intervention developed [99]. Similar measurements have been developed in CF and help determine the effects of clinical interventions on various aspects of daily living (psychological, emotional and social functioning) [100, 101]. Individuals with CF are prescribed treatments that are time consuming and need to be performed several times a day. If these treatments are not perceived as being helpful, they are not performed resulting in low adherence. Additionally HRQOL provides an estimate of an individual's perception of illness severity and how CF impacts his/her life and level of functioning (emotionally, socially, and psychologically). These measures are very useful in determining the impact of new treatments. It is important to perform HRQOL measurements during this study to assess not only the effect of improving nutritional, choline and EFA status in CF, but also to better define the overall impact that Lymxsorb may have on quality of life.

g. Bone Health and Muscle Strength: With patients living longer, low bone density and increased fracture rates are now recognized complications of CF, particularly in older adolescents and adults. Individuals with CF and PI have several risk factors for low bone mass, including poor growth, delayed puberty, malabsorption of calcium and vitamin D, decreased weight-bearing physical activity, and the use of corticosteroid medications [102, 103]. The role of EFA in calcium and bone metabolism is just beginning to be elucidated. In animal studies, fatty acid deficiency has been shown to lead to a loss of bone calcium and matrix, resulting in marked bone demineralization and osteoporosis [104-106]. Furthermore, treatment with omega-3 and omega-6 polyunsaturated fatty acids (PUFAs) has resulted in significant reduction in biochemical markers of bone resorption [104, 106]. Very few human studies have been done testing the effects of PUFA supplementation on bone health, and all have been in older patients at risk for osteoporosis. Two studies showed better bone mass maintenance [107], calcium absorption and stimulation of osteoblastic activity (increased osteocalcin) [108] in subjects receiving PUFA supplements (oils containing linoleic acid, gamma-linolenic acid, eicosapentanoic acid and docosahexanoic acid) compared to those receiving placebo. However, a third study [109] showed no effect of PUFA supplementation on bone mineral density in pre- and post-menopausal women. Further prospective studies of EFA supplementation and bone health in younger subjects are warranted.

From birth to adulthood total skeletal calcium increases from 25 to 1200 grams, manifest as increases in bone mass, dimensions and bone density. Peak bone mass (PBM) is the maximum amount of whole bone mineral content (BMC) attained during the life cycle and a major determinant of the risk for osteoporosis later in life. The exact timing of PBM is unclear; however 90% of PBM is acquired by 18 years of age and 25% is acquired during the two-year period surrounding peak height velocity in the adolescent growth spurt. Therefore, childhood and adolescence are critical periods for the development of lifelong bone health. Physical activity and muscle strength/force, both of which are strongly correlated with bone mineral accretion in children has not been evaluated in children with CF. The fact that fracture rates are not higher in children and adolescents with mild to moderate CF [103] and normal bone mass can be achieved in well nourished people with CF suggests that deficits in bone are not inevitable in CF, and nutritional support and timely treatment of lung infections can optimize early bone mineral accrual [102]. Furthermore, improving EFA status in children with CF may have beneficial effects on calcium and bone mineral metabolism. In this study we will monitor the effects of 18 months of LYM-X-SORB™ supplementation on bone health, using multiple methods and the latest techniques to evaluate bone health including DXA, and pQCT at multiple sites in conjunction with measures of physical activity, muscle strength and force, biochemical measures and markers of bone turnover.

6. Adherence

Adherence to pediatric medical regimens is a significant challenge, with reported rates merely 50% across studies and treatment type [110] and as low as 16% for dietary recommendations in CF [111]. Factors associated with nonadherence to medical regimes in pediatric chronic illness include regimen characteristics (e.g., complexity, negative side effects), disease characteristics (e.g., symptom severity, chronicity), and patient/family variables (e.g., socioeconomic status, psychosocial distress) [112-116]. Assessment of adherence in pediatric research should involve obtaining information continuously and from multiple sources [116, 117], and objective measures of adherence should be used to decrease the bias of parent and child reports [114, 118, 119]. Prior research has demonstrated that behavior modification is effective at improving adherence to nutritional recommendations in CF [120-122], and that increased adherence is maintained at least two years post treatment [121]. Further, this type of behavioral intervention has been effective across disease groups and nutritional targets [123, 124].

In a previous study of LYM-X-SORB™ supplementation in cookies, 10 of the 73 subjects enrolled in the study withdrew reporting adherence difficulty [7]. LYM-X-SORB™ is now provided in a wheat-based flour powder with documented improved palatability and mixing characteristics, and is dissolvable in solid and liquid foods. Considering the adherence difficulties in the prior LYM-X-SORB™ trial and recommendations from behavioral interventions shown to increase adherence to nutritional regimens, we plan to monitor and maximize adherence to LYM-X-SORB™ supplementation by maintaining regular contact with participants throughout the study, quantifying supplement use, gathering parent- and child-report data on supplement use, providing options for supplement consumption, and providing contingency-based incentives for adherence.

7. Stool Microbiome

There is increasing evidence from studies in obesity, inflammatory bowel disease, fatty liver and cancer regarding the importance of the gut microbiome in the pathogenesis of disease. Additionally, changes in the diet composition appear to change the gut microbiome[125] Choline deficiency is associated with fatty liver. It has been shown that a choline deficient diet is associated with changes in the gut microbiome and these changes were associated with changes in liver fat[126]. Patients with CF have fatty liver[17, 42, 44] and abnormal choline metabolism[34, 35] and it is not known if abnormalities in choline metabolism are related to fatty liver in CF. Since the human microbiome is influenced by dietary changes, we wish to see if supplementation with LYM-X-SORB™ will lead to improvement in choline status and so a decrease in fatty liver and whether this improvement is related to changes in the gut microbiome.

PRELIMINARY STUDIES

1. SBIR Phase I Final Report, Avanti Polar Lipids, Inc.

Summary of Specific Aims:

The NIH Small Business Innovation Research Grant 1 R43 DK60302-01 (09/29/01 to 09/29/02) was presented to Avanti Polar Lipids, Inc. in support of evaluating the relationship between structural integrity and palatability in a novel therapeutic lipid matrix for benefiting the clinical well-being of patients with CF. LYM-X-SORB™ is a food product and not a pharmaceutical product. This work was performed in response to the NIDDK interest in "Development of products useful in assessing or improving nutritional status in patients with CF including improvement in pancreatic enzyme preparations".

CF currently affects more than 30,000 people in the USA and Canada and continues to be diagnosed in approximately 1,000 individuals in the US annually. Many of these patients suffer with PI and as a result have difficulty with digestion and intestinal functioning which in turn affects their overall health. Despite enzyme therapy, these patients tend to have compromised fat absorption due to pancreatic limitations and consequently exhibit impaired nutritional and pulmonary fitness [127, 128]. Current treatment for these patients includes, in part, monitoring diet and setting nutritional guidelines high in fat content [2]. A novel lipid matrix, LYM-X-SORB™, composed of LPC, MG and FA has been shown to significantly improve the clinical well-being of patients with CF by providing a readily absorbable fat source [7]. The acceptability of this lipid matrix to the CF community requires a cost effective method of production and, most importantly, palatability.

Production cost containment was maintained by introducing a novel method of PC hydrolysis to yield LPC as well as using an efficient mixing apparatus[129, 130]. In tandem, these improvements led to fast production turn-around in a format capable of a several hundred-fold scale-up. PC hydrolysis was reduced from an original time requirement of several days to 5-6 hours (99+% completion); the preparative process was demonstrated as scalable from a 5-liter batch scale to one 130-liters and greater.

Palatability issues were less manageable. Taste improvements were the guiding concerns behind the grant application and presented work. Good outcomes in prior clinical studies prompted the need to improve LYM-X-SORB™ palatability. It was assumed that the polar head grouping of the lipid matrix is responsible for the undesirable taste inherent to the material currently produced. Stabilizing the polar head grouping and defining the structural integrity of the lipid matrix was examined in order to provide a product with a desirable taste. The specific aims presented in the Phase I grant application were:

1. Determine the structural integrity of a protonated and ionized lipid matrix as a function of water content. It has been assumed that alterations to the physical arrangement of the lipid complex occur with variations to the polar head group state (protonated/ionized) and water concentration. These structural alterations were suggested as a means of changing the taste profile of the lipid matrix to one that is more palatable. The structural integrity of several preparations was evaluated by (1) Low-angle x-ray diffraction, (2) Polarized light microscopy, (3) Freeze fracture electron microscopy and (4) Dynamic light scattering.
2. Define the palatability of the lipid matrix to measurable physical and structural parameters of the intra molecularly stabilized complex. Comparing the palatability of LYM-X-SORB™ (ionized, pH 7.5 and partially protonated, pH 5.5) formulations was performed by sensory evaluation studies at the University of Florida (Gainesville, FL) and Mississippi State University (Starkeville, MS). A fully protonated LYM-X-SORB™ (pH 3.75) formulation was also evaluated by in-house staff (Avanti), some staff of the CF Center at CHOP (Philadelphia, PA), senior staff members of the CF Foundation (Bethesda, MD) and clinical investigator in CF (Montreal, CA).
3. Establish quantitative analytical methods for routine measurement of the lipid matrix and establish specifications that will define and enhance the successful preparation of palatable formulations. Specifically, methods for determining the mole ratio of LPC/MG/FA in the lipid complex, the FA profile, the PUFA content and the moisture content were created.

Protocol/Findings:

Ninety-six LYM-X-SORB™ samples were prepared from synthetic materials. Of those 96, 18 were chosen for initial study as presented in Table 1. The 18 samples were chosen to represent the wide-ranging alterations in head group state and water content. These initial samples were manufactured from synthetic materials to remove the waste and cost of multiple large-scale reactions. The synthetic LYM-X-SORB™ samples were manufactured as 4 different series. For all series, the samples utilized the same LPC (Avanti, Product #845875) and MG (Nu-Chek Prep, Product #M-239) components in the respective molar ratio of 1:4. Variations to the complex head group were then introduced by altering the FA component or salt quantity.

Series 4 represented a fully protonated form of the complex with oleic acid serving as the FA source (Nu-Chek Prep, Product #U-46-A) in a molar ratio of 1:2, LPC:FA. Series 5 utilized sodium oleate (Nu-Chek Prep, Product #S-1120) to represent an ionized head group. The overall LPC:FA molar ratio was maintained at 1:2, however series 5 further divided its samples into various ratios of sodium oleate and oleic acid. Series 6 represented a fully protonated LYM-X-SORB™ in the presence of excess sodium ions and was identical to series 4 in lipid content. Various moles of sodium bicarbonate (JT Baker, Product #3506-05) were added per mole of LYM-X-SORB™ complex to provide the excess ions. Series 8 presented potassium oleate (Nu-Chek Prep, Product #Custom) in lieu of sodium oleate as in series 5. Series 8 was similarly divided into several preparations as in series 5. All ninety-six LYM-X-SORB™ samples were mixed under nitrogen and heated (50°C) to a homogenous oil then capped in a crimp-sealed vial. Various water amounts, from 0 moles to 8 moles per mole of LYM-X-SORB™ complex, were added to each respective sample and mixed to homogeneity. All samples were stored at -20°C.

The preparations in Table 1 were sampled under nitrogen for examination by Karl Fisher water titration, low-angle X-ray diffraction, polarizing microscopy and dynamic light scattering. Freeze fracture electron microscopy (FFEM) was performed on previously prepared synthetic LYM-X-SORB™ mixtures identical to series 4. Karl Fisher water analyses were performed on all 18 samples to verify water content. Low-angle X-ray samples were forwarded to both the Ohio State University and Duke University for evaluation. A Zeiss Axiolab Microscope equipped with polarizer and slide analyzer was purchased for examining samples by polarized light microscopy. The samples were imaged and digitally recorded via an Imaging Planet 1/3" color CCD camera and heated via a Linkam Scientific MC60 warm stage. FFEM samples were forwarded to Nano Analytical Laboratory, San Francisco, CA. Dynamic light scattering (DLS) samples were measured at Avanti Polar Lipids, Inc. with a Brookhaven BI-90 particle-sizer from the laboratory of Barry Sears, Marblehead, MA.
X-ray results provided several insights. The original concept of phase alteration as a function of water content was confirmed; protonated samples with increased water presented a two-phase system as opposed to the lamellar bilayer presented in neat materials. Prior examination of protonated, neat LYM-X-SORB™ displayed 6 reflections that index as the first 6 orders of a lamellar (bilayer) spacing while fully hydrated LYM-X-SORB™ (~8 moles water/mole LYM-X-SORB™) displayed the spacings of two low-angle reflections with the ratio of the square root of three corresponding to the first two orders of a hexagonal phase. X-ray data demonstrate that water in the protonated samples (Series 4) altered the lipid bilayer and presented a second phase. Sharp continuous X-ray rings consistent with a lamellar bilayer were replaced by crystalline spacings of another structure. This second phase was consistent with the hypothesis that an inverse hexagonal structure results with increased water. Identification of the inverse hexagonal phase, however, could not be made by X-ray diffraction. The conversion from a low-water structure to a high-water structure was visualized by polarized light microscopy. Where regions of homogenous striations in 4.A. were only partially disrupted by non-conforming crystallinity, sample 4.F. presented no such uniform striations.

In the presence of sodium, water had minimal effect. X-ray data show that sodium, whether in the form of sodium bicarbonate in a protonated matrix (Series 6) or sodium oleate as the FA constituent (Series 5), stabilized the bi-layer regardless of water content. The characteristic bands of sharp continuous X-ray rings were noted in both low- and high-water samples indicating the phase withstood hydration. Potassium ions had a similar but opposite effect. Potassium oleate as the FA constituent (Series 8) demonstrated crystalline spacing regardless of water content. None of the ionized series presented the uniform striations seen in the

low-water protonated samples via polarizing microscopy. The crystallinity of these matrices did not appear to alter as a function of water content or salt form. Ionized samples presented similar polarization patterns regardless of salt form, salt amount or water content.

Polarizing microscopy was performed on all samples in Table 1. All samples were spotted on a microscope slide and covered with a 22x40 mm cover glass for examination. Each sample was then placed on a warm stage to melt and imaged upon cooling to ambient. Melting temperatures varied greatly with series. All samples in series 4 and samples 5.G. to 5.L. melted prior to 60°C. The remaining samples required temperatures of up to 150°C to fully melt. Significant differences in the polarized images as a function of water content or ion presence, excluding the samples in series 4, were not noted. FFEM images of a protonated synthetic LYM-X-SORB™ collaborated with X-ray results and further demonstrated a structural change as a function of water content. In the low water sample, large bilayer vesicles are quite intact and display few sponge-type structures at the contact points of undulating bilayers. In a high water sample, the number of contact points and subsequent sponge-type structures increases. While a structural change as a function of water was noted, positive identification of the resulting phase could not be made. Dynamic light scattering measurements were made of all samples in Table 1 for size determination. All samples were prepped in water and measurement made with a minimal instrument rate count of 40 Kcps. Instrument accuracy was verified by known polystyrene standards of 90 and 304 nm (Ladd Research, Williston, VT). All measurements were made in triplicate and the results demonstrated a trend in particle size between protonated and ionized samples. In order from largest to smallest, the general trend in particle size was 8.S-X > 5.S-X > 6.S-X > 5.G-L > 8.G-L > 4.A-F. Two moles of potassium oleate as the FA constituent had the largest average particle while the protonated Series 4 had the smallest. The trend suggested that particle size was a function of salt form and degree of ionization. Protonated samples, with no bulky salt ion present, were subsequently the smallest.

Table 1: Synthetic LYM-X-SORB™ Sample Identification

Sample ID	Mole Ratio				Variant, X	Water Content
	LPC	*MG*	*FA*	*X*		*Moles*
4.A	1	4	2	0	None	0.0
4.C	1	4	2	0	None	3.0
4.F	1	4	2	0	None	8.0
5.G	1	4	1	1	Sodium Oleate	0.0
5.I	1	4	1	1	Sodium Oleate	3.0
5.L	1	4	1	1	Sodium Oleate	8.0
5.S	1	4	0	2	Sodium Oleate	0.0
5.U	1	4	0	2	Sodium Oleate	3.0
5.X	1	4	0	2	Sodium Oleate	8.0
6.S	1	4	2	3	Sodium Bicarbonate	0.0
6.U	1	4	2	3	Sodium Bicarbonate	3.0
6.X	1	4	2	3	Sodium Bicarbonate	8.0
8.G	1	4	1	1	Potassium Oleate	0.0
8.I	1	4	1	1	Potassium Oleate	3.0
8.L	1	4	1	1	Potassium Oleate	8.0
8.S	1	4	0	2	Potassium Oleate	0.0
8.U	1	4	0	2	Potassium Oleate	3.0
8.X	1	4	0	2	Potassium Oleate	8.0

Defining the palatability of the lipid matrix to measurable physical and structural parameters was done via modification to production-scale LYM-X-SORB™ preparations. Results from the initial synthetic materials suggested that the degree of ionization had a greater impact on structural arrangement than did water content when sodium was present. Production-scale LYM-X-SORB™ reactions where PC hydrolysis is performed rely on sodium bicarbonate as a necessary reaction buffer, thus the elimination of sodium was not an immediately viable option. Modifying the ion content in the matrix itself was performed to test the palatability of protonated versus sodium-ionized batches with similar water contents (interrupted bi-layer versus salt-stabilized bi-layer).

Three, two-kilogram LYM-X-SORB™ batches (Avanti reaction numbers 5.64, 5.65 and 5.66) were manufactured under identical conditions. Prior work had established the constituent materials and ratios best suited for a CF population and these were adhered to as previously manufactured with the overall LPC:MG:FA ratio in all batches remaining 1:4:2. Raw materials consisted of LPC from hydrolyzed soy PC (Natterman,

Product #8729), monoglycerides of varying acyl saturation and composition (Danisco, Products #U/D-KA, #S-KA, #TS-ED 205) and fatty acids of varying acyl saturation and composition (Nu-Chek Prep, Product #N-16-A; Cognis/Henkel, Product #790). Production was maximized utilizing the referenced manufacturing improvements for cost containment [129, 130]

Batch reaction 5.64 was manufactured and discharged as an ionized mix, sodium bicarbonate acting as the constituent salt source and a final moisture content of 0.63%. [Represents ~1.0 mole water/mole LYM-X-SORB™ (see Table 1, samples 6S, 6U).] The pH was measured as approximately 7.5. Batch reaction 5.65 was manufactured and discharged as a partially protonated mix, phosphoric acid being titrated in to a pH of 5.5 prior to discharge. The final moisture content was 0.88% or ~1.0 mole water/mole LYM-X-SORB™. A pH of 5.5 was identified as a level at which approximately half of the fatty acids would be protonated (see Table 1, samples 5G, 5I). Batch reaction 5.66 was manufactured and discharged as a fully protonated mix, phosphoric acid titrated in to a pH of 3.75 prior to discharge with a final moisture content of 0.75%, or ~1.0 mole water/mole LYM-X-SORB™. Each material was then incorporated into butter cookies as a substitute for shortening (16% dry wt. per cookie); the cookie recipe provided by permission from Bud's Best Cookies (Hoover, AL). All cookies were manufactured under identical parameters and stored at ambient temperature. Each cookie measured 40 mm (1.5") in diameter and was between 4 mm (0.125") and 6mm (0.25") thick and contained ~0.55g LYM-X-SORB™ or shortening respectively. Cookies utilizing LYM-X-SORB™ from reaction 5.64 were labeled as Series B (ionized), cookies utilizing LYM-X-SORB™ from reaction 5.65 were labeled as Series A (partially protonated). Cookies utilizing LYM-X-SORB™ from reaction 5.66 were labeled Series 3.75 (fully protonated). A set of control cookies was manufactured LYM-X-SORB™ utilizing the shortening component as originally called for by the recipe and labeled Series C. Samples of cookie sets A, B and C were forwarded to the University of Florida and Mississippi State University for sensory evaluation studies. Cookies from series 3.75 were submitted to in-house staff (Avanti), staff of the CF Center at CHOP (Philadelphia, PA), senior staff members of the CF Foundation (Bethesda, MD) and a clinical investigator in CF.

Palatability results between the universities collaborated well, albeit not as anticipated. One hundred panelists from the University of Florida and one hundred and fifty-nine panelists from Mississippi State University participated in the study. Each panelist was provided three of each cookie series and asked to rate the cookies in aroma, flavor, mouth-feel, aftertaste and overall acceptance on a 9 or 10-point hedonic scale (with the highest score, the best tasting) (Table 2). Orange juice was provided as a palate cleaner between cookie sets. In all areas, the control cookie (C) was clearly distinguished from the LYM-X-SORB™ cookies by a statistically significant margin. Cookie series A and cookie series B were relatively indistinguishable from one another, however. Cookie C rated well above the experimental cookies in overall acceptability and provided the most favorable comments. Cookies A and B both received lower scores based on their minimal aftertaste and mouth-feel however both cookies were deemed palatable.

Table 2: Cookie Overall Acceptability Ratings

	A - LXS	B - LXS	Control
University of Florida (9-pt scale)	2.8	2.9	5.8
Mississippi State University (10-pt scale)	3.2	3.5	5.6

The palatability of cookie Series 3.75 (fully protonated) was very favorable. The in-house, CHOP, CFF and clinician evaluations were equally encouraging; all participants judging the fully protonated LYM-X-SORB™ cookies acceptable. The small cookie size and taste improvement offered by protonated LYM-X-SORB™ overcame initial concerns of cookie quantity proposed as a LYM-X-SORB™ dosage (30 cookies/day) for patients with CF. Evaluation comments were positive; notes provided by clinician review concluded that the cookies would be acceptable to patients with CF of all ages. The in-house staff has further evaluated the palatability of the powdered ionized LYM-X-SORB™ (pH 7.5) with acidic foods; such as, SlimFast, orange juice, V8 juice and Dannon Yogurt and found them to have acceptable taste. The total acidity of these foods was important to the quantity need for acceptable taste. Mixing ionized LYM-X-SORB™ with milk (basic), for example, is not recommended.

Material release criteria for palatable formulations were established by quantitative analytical measurements. LYM-X-SORB™ analytical methods were established for lipid ratio confirmation by HPLC, fatty acid profile by GC/FAME, poly-unsaturated fatty acid content by GC/FAME and moisture content by Karl Fisher water titration. Analysis of LYM-X-SORB™ production batches allowed for analytical method development and establishment of standard operating procedures where applicable. Using these methods in conjunction with information from the synthetic LYM-X-SORB™ structural evaluation and palatability results of production batches 5.64 5.65 and 5.66, a final product specification list was established (Table 3).

Table 3: Specifications Criteria for LYM-X-SORB™ Release

Analysis	Specification	SOP# (Avanti)
Physical Examination	Amber solid at room temperature<br>Amber oil at >50°C	200036
HPLC (Mole Ratio)	LPC: 0.95 – 1.25; MG: 3.5 – 4.5; FA: 1.5 – 2.5; MG+FA: 5 - 7	200062
GC-FAME (FA profile)	n6/n3: 4.8 - <5.5<br>n6+n3: > 48%	200002
Karl Fisher (Water Content)	< 2 %	200006
MDA (Oxidation)	< 90 nmoles/g	-
PH	7.0 – 7.5	-
Heavy Metals	< 20 ppm	-
Arsenic	< 10 ppm	-
Microbial	< 100 cfu/g total yeast, molds; < 100 cfu/g total aerobic bacteria<br>S. Aureus, Salmonella, E. Coli, P. Aeruginosa (all negative)	USP Method #61

Results Summary, Discussion and Relevant Progress:

Structural alterations of the LYM-X-SORB™ head group state as a function of ion/water content were demonstrated by X-ray diffraction, polarized light microscopy and freeze fracture electron microscopy. Results show that in protonated samples water created a phase de-stabilization yielding a proposed inverse hexagonal structure (Illustration 1). Salt ionization, on the other hand, stabilized the respective head group phase and minimized the effects of water. Salt form also had an important influence on structure and size. The larger potassium salt was a significantly larger complex than sodium and produced a separate structural phase.

Palatability enhancement as a function of these observed physical alterations was demonstrated. Review of the sensory evaluation data shows that palatability of the ionized (basic, pH 7.5) or partially protonated (pH 5.5) polar head groups were partially compromised, whereas fully protonated LYM-X-SORB™ (pH 3.75) was acceptable. While differences in structure were noted for various FA forms and water concentrations, taste improvement was found to be associated with the protonated LYM-X-SORB™ only. Many in-house sensory trials consistently produced data that suggested taste improvement was obtained by protonating the matrix or mixing with the sufficient amount of acidic foods. Multiple masking attempts rated protonated LYM-X-SORB™ preparations as a blended material in several food/beverage matrices as acceptable.

Analytical methodologies were established and verified the lipid complex. Heavy metal content, arsenic and microbial limits were not established. A third party contractual service quantified these limits. Quantitative measures for water content and oxidation level were established. Quantifying taste attributes in a lipid matrix, especially one that includes hydrolysis products, was a broad task. Complex head state alterations can influence palatability. A definitive link between known structural parameters and taste was identified and several parameters were eliminated. The information gained from structural data does present relevant progress in assessing the relationship between the matrix organization and taste. Establishing this relationship is warranted because LYM-X-SORB™ as a medical food has improved the clinical well-being of patients with CF and has a potential use in AIDS, cancer, short bowel syndrome and the elderly. Improving these product and taste ratings through further refinements between structure and pH remained the focus after Phase I. This new generation formulation LYM-X-SORB™ in wheat and sugar powder, was developed, and results of palatability testing are summarized in Preliminary Studies.

3. Qualifications of the Investigators

The research team is a unique group of accomplished scientists with the experience needed to execute this study. **Dr. Walter A. Shaw** is founder and president of Avanti Polar Lipids, Inc. He has developed Avanti into a company that produces many unique lipid molecules for the researcher and a company that produces cGMP grade lipid products for pharmaceutical applications. Many of Avanti's products are used as excipients and /or active ingredients in FDA approved drug formulations. The leadership role Avanti plays in the lipid industry has been validated as they were selected to join the LIPID MAPS consortium as the supplier of lipid standards. Avanti currently holds grants and contracts from the National Cancer Institute to produce lipid like molecules and the lipid based oral drug delivery vehicle, LYM-X-SORB™, complexed with drugs to be used in human clinical trials. **Dr. David Yesair** is founder and President of BioMolecular Products, Inc. and its four LLC subsidiaries. He is the inventor of the LYM-X-SORB™ platform technology that includes its composition of matter, multiple uses and manufacture. Dr. Yesair, together with licensees of this technology has participated in the safety and efficacy evaluation of LYM-X-SORB™ in both animals and humans and has established the chemical/physical characteristics of LYM-X-SORB™. In collaboration with Dr. Claude Roy (Montreal), he was the PI of a SBIR Phase I grant that established the readily absorbable characteristics of oral LYM-X-SORB™. He was also a member of the same research team that evaluated the beneficial effects of LYM-X-SORB™ on lipid absorption and the clinical outcome in patients with CF [7]. He developed the general objectives of the present SBIR application and enlisted the collaboration of both Drs. Virginia Stallings and Walter Shaw in participating in this innovative clinical program. **Dr. Jeff D. Moore** is the director of analytical technologies at Avanti Polar Lipids, Inc. He has extensive experience in the analytical aspects of lipid and phospholipids. His expertise in mass spectrometry techniques is utilized in the quality control of Avanti's many lipid products, provided mass spectral analysis of phospholipids and related compounds from numerous matrixes to researchers and directs Avanti's analytical and reference standard efforts to the LIPID MAPS consortium. He has published in the area of small molecule quantitation by HPLC/MS/MS which is essential to monitoring lipid changes in patients with CF. **Michael Lee Roberts** is currently the process engineer at Avanti since 2004. Prior to this he was the supervisor and director of cGMP Manufacturing at Avanti. He has extensive experience in the manufacturing of lipid compounds.

Dr. Virginia Stallings is a Professor of Pediatrics at University of Pennsylvania School of Medicine, Director of the Nutrition Center at CHOP, and the Deputy Director of the Stokes Research Institute at CHOP. She was the principal investigator of several NIH and Foundation funded research projects, including an NIH multicenter CF longitudinal study, "Nutrition Status and Progression of CF Pulmonary Disease", and two recent CF Foundation sponsored grants, “Bone Health in People with Cystic Fibrosis”, and “Malabsorption Blood Test: A Novel Approach to Quantify Steatorrhea.” Dr. Stallings is also a co-investigator of several other NIH-funded clinical studies. She has conducted pediatric clinical nutrition research for the last 20 years including research in CF. **Dr. Maria Mascarenhas,** Co-Investigator, is the Assistant Director of the CF Center, the Chief of the Nutrition Section at CHOP, and an Associate Professor of Pediatrics at the University of Pennsylvania. For the past four years Dr. Mascarenhas has collaborated with Drs. Stallings and Schall on the CF Foundation-funded project “Malabsorption Blood Test: A Novel Approach to Quantify Steatorrhea.” In addition to her experience in clinical nutrition, pediatric gastroenterology and CF, she has been a co-investigator in several pediatric clinical research trials. **Dr. Asim Maqbool**, Co-Investigator, is an Assistant Professor of Pediatrics and a faculty member of Gastroenterology, Hepatology & Nutrition at CHOP and at the University of Pennsylvania, having completed his GI and Nutrition Fellowship in 2003, Dr. Maqbool has been involved with CF and Nutrition research at CHOP and performed the palatability testing study of the second generation of LYM-X-SORB™ at CHOP. His more recent research endeavors include investigating essential fatty acids deficiency and its correlates in young children with cystic fibrosis. **Dr. Kevin Hommel’s** expertise is in the area of behavior modification to improve adherence to pediatric medical regimens, and has particular knowledge of adherence to nutritional recommendations. In two recent studies at Cincinnati Children’s Hospital Medical Center, Dr. Hommel served as manager and lead treatment provider for 8-week behavioral interventions to increase dietary calcium intake in children with inflammatory bowel disease (IBD) and juvenile rheumatoid arthritis (JRA) [123, 124]. The findings of these investigations underscore the utility of behavioral intervention to improve medical regimen adherence in pediatric populations, as well as the relevant expertise of the study team***.*** **Dr. Joan Schall**, Co-Investigator, is Associate Director of the Nutrition and Growth Laboratory at CHOP, and has been involved in child growth, development and nutrition research for the past 20 years. Her area of expertise is

growth, nutritional status, body composition and energy expenditure in healthy children and children with chronic diseases, including CF. Dr. Schall has been a co-investigator in two CF Foundation-sponsored grants, “A Novel Approach to Quantify Steatorrhea”, and “Bone Health in People with Cystic Fibrosis.” She has also worked extensively on the NIH grant, “Nutritional Status and Progression of CF Pulmonary Disease”, for which Dr. Stallings was the P.I. She is an expert anthropometrist, has experience with the assessment of resting energy expenditure and trial oversight, and has published papers and conducted training sessions in growth assessment. **Dr. Marci Pelchat**, Consultant, is a Senior Scientist at Monell Chemical Senses Center in Philadelphia and a specialist in the field of Food Science and Taste. She has collaborated with the CHOP team in the palatability study of LYM-X-SORB™ in subjects with CF, which has provided preliminary data for the present study. Over the course of the study, Dr. Pelchat will continue to advise the team in developing the best food delivery system for the LYM-X-SORB™, to help ensure palatability and adherence to the supplementation protocol.

4. Research Studies in CF at CHOP

Dr. Stallings has had successful experience with collaborative research in studies of CF at CHOP over the past 20 years. Her current leadership in the nutrition and CF field is reflected by her past or current membership in the CF Foundation Clinical Research Advisory Committee and participation in various NIH, FDA, and CF Foundation conferences on nutrition issues in CF. She was also instrumental in crafting the most recent consensus guidelines from the CF Foundation for maintaining optimal growth and nutritional status in subjects with CF [131]. She currently leads a CF Foundation and National Library of Medicine project to develop the next set of nutritional guidelines for people with CF. The CF research team at CHOP, under Dr. Stallings leadership, has conducted many studies. These include: 1) a four-year longitudinal study of pre-pubertal children with CF to examine the relationship between growth, dietary intake, body composition, energy expenditure and pulmonary function [132-137]; 2) a study of maturity status and REE in adolescent girls with CF compared to a group of healthy controls [138]; 3) A longitudinal study of REE in children with CF taking a pulmonary inhalation medication (rhDNAse) [139]; and 4) a study to determine prospectively the relationship among growth, nutritional status and pulmonary function for four years using the CF Foundation Patient Registry Data [140]. The recent CF research at CHOP has included: 5) an NIH-sponsored collaborative multicenter study (13 CF centers) of 91 pre-pubertal children with CF and PI, followed for two years, to determine the role of growth and nutritional status as prognostic indicators of pulmonary function (dietary intake, growth and nutritional status including serum EFA and fat-soluble vitamin status, pulmonary function status, REE, total energy expenditure, fecal fat malabsorption, and exercise performance) [141]; 6) a CF Foundation-sponsored study of bone health in 100 people with CF (ages 8 to 25 years), and the relationships among growth, nutritional and pulmonary status and bone health using comprehensive measures of bone status [103]; and 7) a CF Foundation-sponsored study to develop a malabsorption blood test to replace the 72-hour stool collection method for determining degree of fat malabsorption. The CF research team at CHOP has the breadth and depth of experience to conduct successful collaborative research in the nutritional care of subjects with CF.

5. Summary of LYM-X-SORB™ and Palatability Studies

The first generation/formulation of LYM-X-SORB™ was evaluated in a trial where the LYM-X-SORB™was provided in a wafer cookie by creaming LYM-X-SORB™ with sugar and served as two cream layers between three wafer layers. The cookie had marginal palatability and subjects developed taste fatigue which contributed to a suboptimal compliance pattern. The Avanti SBIR Phase I study was to formulate a more palatable product. The SBIR Phase I did produce a more palatable cookie. These SBIR I results prompted us (Avanti, LXS, LLC, CHOP) to further develop the product as LYM-X-SORB™ oil dispersed into wheat flour and granulated sugar that could be mixed with a variety of foods. This second generation/new formulation, referred to as LYM-X-SORB™ powder, will allow subjects the flexibility to select and change the food/beverage with which to mix the powder. This minimizes the taste fatigue. In collaboration with the Monell Chemical Senses Center (Philadelphia PA), a formal palatability study with 34 subjects with CF was performed on the LYM-X-SORB™ powder (Table A). The study results identified eight commercially available commonly consumed food that serve as excellent vehicles for the LYM-X-SORB™ powder in the proposed age range.

Table 4: LYM-X-SORB™ Palatability Studies[a] *as % of subjects*

%Rating	Peanut Butter&Jelly	Chocolate Candy	Grape water ice	Orange juice	Oatmeal	Apple sauce	V8 Citrus Splash	Strawberry yogurt
Good	50	56	32	32	38	26	24	15
OK	29	23	44	35	29	24	35	41
Bad	21	21	24	32	32	50	41	44
Overall Ranking[b]	3.9±2.3	3.2±2.1	4.0±2.2	4.4±2.2	4.5±2.6	5.6±1.9	5.0±2.1	5.5±2.1

[a] Data obtained from 14 male and 20 female subjects ages 9 to 22 (*M* = 15) recruited from CHOP CF Clinic.
[b] The overall ranking of the foods, based on mean rank with 1 = Best and 8 = Worst.

Briefly, 32 g LYM-X-SORB™ powder (standard dose) was added to one serving of each food. The serving was then divided into smaller samples for the subjects to taste. The method of Birch [142] was used to assess food preference. This method is successful in children and has been shown to be both a reliable and valid measure of food preference/palatability [143, 144]. Each subjects took part in one sessions during which they rated all eight foods. Foods were presented one at a time in random order and identified as good, ok, or bad. Subjects were given a pretzel to eat and water to drink between samples. Each food was presented one at a time, in a random order. Subjects were told to taste each sample and place the food on a smiley face that was happy (if they liked the way the taste), neutral (if the taste was just "ok," not good or bad), or sad (if they disliked the taste). For the foods placed on the happy face, subject were asked which of those foods he/she liked the very best. The experimenter removed the food that the subject indicated and asked the question again until all of the foods were removed from the happy face. The procedure was hen repeated with the foods on the neutral and sad faces. This yielded an eight-point ranking of the foods with lower numbers indicating good palatability. The results of this study demonstrated that the LYM-X-SORB™ powder produced a highly palatable product. As expected, there were individual differences in liking for each of the foods, however, each of the subjects found some foods to be good or OK (acceptable); 32 out of 34 subjects (94%) rated at least one food as good and all subjects placed at least three of the foods in either the good or the ok category. Sex and age did not affect palatability ratings. The most successful vehicles were peanut butter & jelly sandwich, chocolate candy, oatmeal and grape ice.

While palatability is not the only predictor of long-term compliance, it is a necessary component. There is evidence that food preference/palatability in children is highly correlated with consumption [144, 145]. Not only does the wheat flour/sugar LYM-X-SORB™ powder significantly improve the palatability, it also provides a framework that allows the study subjects the freedom to select and change the food/beverage used for delivery. This will eliminate the taste fatigue in most participants, and adherence is expected to be excellent in this proposed LYM-X-SORB™ study.

6. Summary

The 12-month supplementation study in Canadian subjects [7], showed an improvement in EFA status and growth parameters. A trend towards fewer hospital admissions were seen at 18 months, showing a persistent effect six months after supplementation ended. In the Canadian study, taste and palatability were factors that influenced adherence and the drop out rate. Our study will differ from the previous study in that we will supplement subjects with CF for 18 months and study the effects on different organ systems and metabolism, and will be using the new palatable LYM-X-SORB™ powder mixed in a number of age-appropriate, frequently consumed foods and beverages. The new product (lipid/flour/sugar) has better mixing characteristics and improved palatability. The proposed study extends the study of the effects of LYM-X-SORB™ from EFA to choline status, metabolic changes in the body, inflammatory cytokine levels, bile composition, hepatic steatosis, REE, RQ and effects on clinical course. If successful, this study will prove the many beneficial effects of LYM-X-SORB™ and will be quickly incorporated into care to improve the care and well being of patients with CF.

RESEARCH DESIGN AND METHODS:

This study will consist of three phases. **Phase I**: Subject screening, enrollment and recruitment: subjects will be screened for eligibility at each study site CF center. Local CF center coordinators will be requested to pull charts of all patients with CF currently followed at their study centers who meet the following criteria: pancreatic insufficiency, ages 5-18.9 years and FEV1 greater than 40% predicted. Charts will be screened by research study personnel for study eligibility. The study personnel has a waiver of consent to screen approximately 50-80 charts at each site. Subjects will not be eligible for the study if they have an FEV_1 <40% predicted (determined from medical records), residual pancreatic lipase activity (screening fecal elastase >15 ug/g stool), or evidence of significant liver disease (GGT >3x normal from medical records). See Table 5. Subjects will be recruited from nine CF centers: Children's National Medical Center, Washington, DC; Children's Hospital of Philadelphia, Philadelphia, PA; Monmouth Medical Center, Long Branch, NJ; The Pediatric Lung Center (Fairfax Neonatal Associates, PC), Fairfax, VA; Cystic Fibrosis Center of University of Virginia, Charlottesville, VA; Children's Hospital of the King's Daughters, Eastern Virginia Medical School, Norfolk, VA; Yale University School of Medicine, New Haven CT; Schneider Children's Hospital, North Shore Island Jewish Health System, New Hyde Park, NY; St. Joseph's Children's Hospital, Paterson, NJ; and The Pediatric Specialty Center at Lehigh Valley Hospital, Bethlehem PA. **Phase II:** Supplementation phase for primary outcomes: subjects will be seen at the baseline visit and randomized to receive supplementation with either LYM-X-SORB™ or placebo along with coupons for food items used to deliver the supplement. They will be seen at CHOP for the major protocol visits at baseline, 3, 12 and 18 months, while the 6-month visit will take place at the subject's home CF Center. The 12-month visit is the primary endpoint to determine the effect of LYM-X-SORB™ on EFA and choline status. **Phase III:** Continued supplementation for secondary outcomes: subjects will continue to take either LYM-X-SORB™ or placebo through 18 months, the time of the final protocol visit. In addition to determining the longer-term effect of LYM-X-SORB™ vs. placebo on EFA and choline status, the effect on pulmonary status and growth status will be determined at the 18-month visit.

The primary aim of this study is to determine if EFA and choline status, as measured by linoleic acid and the triene/tetraene ratio, and the PC/PE ratio, are improved by supplementation with LYM-X-SORB™ over a twelve month period in subjects with CF and PI, ages 5.0 to 18.9 years, using a randomized, double blind, placebo-controlled research design. Secondary aims are to determine if LYM-X-SORB™ supplementation will also improve growth, nutritional, and pulmonary status over an 18-month period. Subjects (n=112) will be recruited for the study and randomized to receive supplementation with either LYM-X-SORB™ or placebo in a variety of foods and beverages for 18 months.

Table 5. Enrollment Criteria

Inclusion Criteria at Enrollment	Exclusion Criteria at Enrollment
• Cystic fibrosis with pancreatic insufficiency<br>• Age: 5.0 to 18.9 years<br>• In usual state of good health<br>• Family and subject commitment to the18-month study protocol<br>• Fecal elastase < 15 ug/ g stool	• FEV_1 < 40% predicted<br>• Other chronic health conditions that may affect GI absorption, growth, dietary intake, nutritional status<br>• Liver disease, lung transplant, celiac disease, allergy/intolerance to wheat/gluten, pregnant<br>• Participation in another CF nutrition-related intervention study<br>• Fatty acid (i.e. fish oils) or choline supplements<br>• Home parenteral lipid administration (i.e. intralipids)

1. Subject Inclusion Criteria (Table 5): All subjects will be enrolled in their usual state of good health defined as no hospitalizations, emergency room or unscheduled acute illness clinic visits, and with activity levels and food intake considered typical by the subject and their care provider for two weeks prior to enrollment. Because the LYM-X-SORB™ is supplied in a wheat-flour based powder, subjects with celiac disease or allergies or intolerance to wheat or gluten will not be eligible. Subjects who become pregnant during the course of the study will not continue participation. Subjects taking any nutritional supplements

containing EFAs, such as fish oils or intralipids, or choline supplements are not eligible for the study. Subjects who are willing to discontinue taking these supplements for the duration of the study will be eligible after a two-month period off the supplements, with the approval of the subject's CF physician.

2. Subject Recruitment and Enrollment: Subjects will be recruited from ten CF Centers. Subjects will be recruited via telephone to be invited to participate in the study. This requires an enrollment rate of 18% of the approximately 445 subjects ages 5.0 to 18.9 available at the Centers combined. Based upon previous experience, we will achieve this without difficulty.

3. LYM-X-SORB™ Supplement: Subjects will be provided with either the LYM-X-SORB™ or placebo powdered supplement and with a variety of food and beverage coupons at each major protocol visit. (baseline, 3, 6, and 12 months). In all other months, the supplement will be shipped directly to subjects and families. LYM-X-SORB™ supplement will be provided in sealed packets, each with 6 g of LYM-X-SORB™in approximately 32 g of powder (the total weight of the powder for each serving may vary from 26 to 37 grams, but will be 32 grams on average). Subjects will add the LYM-X-SORB™ or placebo powder to food before ingestion two or three times each day in a size-dependent dose of approximately 24 g/1.72 m^2 [7]. Subjects between the ages of 5.0 and 11.9 years will add 12 g/day of LYM-X-SORB™ (2 packets per day of 6 g each), and subjects ages 12.0 to 18.9 years will add 18 g/day (3 packets per day of 6 g each). Using the CDC 2000 growth charts [146], the 50th percentiles for weight for each age group range from 21 to 40 kg in males and 20 to 42 kg in females for the 5.0 to 11.9 year old age group, from 40 to 67 kg for males and 42 to 56 kg for females in the 12.0 to 18.9 year old age group. Supplements will be shipped to participants' homes each month and at the end of each month, families will be contacted to record the number of unused packets from the previous month.There will be coupons for a variety of food and beverage choices in order to avoid taste fatigue and enhance adherence. The initial food choices will be suggested from the palatability study and from subject preference. All research personnel responsible for collecting data on the subjects will be blinded to the randomization throughout the study. Study supplement will be labeled with a code number. Each monthly supply of supplement will contain 10% overage. Two individuals at CHOP will be designated to have access to the randomization code. These two individuals will include a member of the Pharmacy staff and a physician trained in nutrition research and care who will not participate in any aspect of the study. In the event of a medical emergency, Dr. Stallings will be unblinded for that individual subject so that she can provide the information to the subject's care team at CHOP or at their home CF Center.

4. Protocol, Schedule and Reimbursement Schedules: We will reimburse subjects/families at a standard rate to offset incurred expenses for travel, food, and babysitting expenses. Subjects/families will receive $100 for the baseline, 3 and 12 month visits as the overnight visits, and also at 18 months, the final visit, all of which will occur at CHOP. They will receive $50 for the 6-month visit at their own CF Center. The total reimbursement for subjects will be $450. In addition, subjects will be given an age-appropriate gift card for book/DVD/video/CD (average value = $25) at each month interval as a thank-you for participating in the study and continuing to take the supplement. The amount of the gift will be contingent upon the number of points the subject receives for taking the supplement each month (1 point per day worth $1/day if they take the supplement). We anticipate an adherence rate of ~80-85% corresponding to a monthly gift of $25 on average for each subject. In addition to the flat rate of reimbursement developed for subjects from the Philadelphia area, the cost of long-distance transportation will be reimbursed for your child and one family member to accompany your child to CHOP.

5. Study Design: Table 6 provides a summary of measurements for the five protocol visits. A timeline for the study is provided in the Budget Justification for CHOP.

Table 6. Summary of Methods and Visits (n=112)

	Screening	Baseline	3 mo	6 mo	12 mo	18 mo
**Visit to CHOP (*overnight admission)**		√*	√*		√*	√
**Visit to Home Center**				√		
**Lipid Status and Serum Vitamins**						
Serum Fatty acid profile, lipid panel, plasma sterols, vitamins A, E, D and K		√	√	√	√	√
Fecal fat		√	√		√	
**Choline Status**						
Serum choline, phosphatidylcholine (PC), phosphatidylethanolamine (PE), homocysteine, methionine, lysophosphatidylcholine, vitamin B_6, B_{12}, and acyl carnitine		√	√	√	√	√
RBC folate		√	√	√	√	√
Fecal phosphatidylcholine		√	√		√	√
**Metabolic, Inflammatory, and Infection Assessments**						
Pulmonary function test (Spirometry), Respiratory muscle strength		√	√		√	√
Sputum culture		√				√
Bile acids, serum and urine		√			√	
Magnetic resonance imaging and spectroscopy (MRI/MRS) of liver**		√			√	
C reactive protein		√	√		√	
Resting energy expenditure/Respiratory Quotient		√	√		√	
DXA (whole body for body composition)		√	√		√	√
**Safety, Growth and Dietary Intake**						
CBC with differential, urinalysis, blood cyanide		√	√	√	√	√
Complete metabolic panel		√	√	√	√	√
Serum Gammaglutaryl Transferase (GGT)		√	√	√	√	√
Genotype (20%)		√				
Fecal elastase	√					
Height, weight, skinfolds, circumferences		√	√	√	√	√
Sexual maturity status (pubertal and menarcheal status)		√		√	√	√
**Bone mineral density and content: DXA, pQCT**		√			√	√
**Bone markers in serum: osteocalcin, iPTH, fractionated alkaline phosphatase**		√	√		√	√
**Muscle Strength assessments: BIODEX, Force plate, Hand grip dynamometer**		√	√		√	√
3-day weighed food intake		√	√		√	√
**Other Assessments**						
Medical Record Review	√	√	√	√	√	√
Adherence to Supplement Survey		√	√	√	√	√
Disease Severity Assessment		√	√	√	√	√
Adverse Events Diary		√	√	√	√	√
Multiple Family and Health Questionnaires		√	√	√	√	√
Quality of Life Questionnaires (PedsQL 4.0, CF-QOL)		√		√	√	√
Stool analysis for microbiome		√	√		√	

*Visits by subjects to CHOP will take place at Baseline, 3, 12 and 18 months. Project staff will visit subjects at their home centers at the 6 mo visits. Monthly supplies of LYM-X-SORB (active or placebo) will be shipped directly to subjects' home each month. **The MRI/MRS will be performed on a subset (n=24) of subjects 10 years of age or older at baseline and 12 months.

6. Methods and Measurements

a. Essential Fatty Acid and Choline Status Assessment: We will use high performance liquid chromatography coupled with triple quadruple mass spectrometry (HPLC/MS/MS) and capillary gas chromatography with flame ionization detector (GC/FID) to quantitatively analyze (i) the choline pool balance, (ii) changes in choline related phospholipid classes and (iii) fatty acid profile of the serum total lipid extract including free acid and esters derived from the lipid parent structures. All methods are performed using Good Laboratory Practices standards. HPLC/MS with selected ion monitoring will be used to quantify water-soluble choline metabolites, i.e.; choline, betaine, acetylcholine glycerophospho- choline, phosphocholine, and cytidine diphosphocholine from both murine and human extracts [147]. The reported sensitivity and quantitative precision is excellent. A similar HPLC/MS method will be employed in this study to measure the water soluble (serum) choline compounds, excluding acetylcholine. The presence of cholinesterases in serum samples will negate the usefulness of measuring acetylcholine levels.

PE, PC, sphingomyelin (SM), and LPC are readily measured by HPLC/MS/MS techniques [148-151]. The total lipids of serum and stool samples will be initially extracted by the method of Bligh and Dyer [152]. Rather than separate and purify each class of phospholipid prior to infusion into the MS interface, a portion of the lipid extract will be subjected to solid phase extraction to remove all neutral lipids [153]. The phospholipid extract is then injected on a normal phase HPLC column connected to the MS interface allowing direct measurement of the different molecular species of each phospholipid using the MS/MS techniques of precursor ion and neutral loss monitoring. In these experiments, PE molecules fragment, resulting in loss of the phosphoethanolamine headgroup, i.e. a neutral loss of 141 u. The PC, SM and LPC fragment to form a common 184 u cation. This corresponds to the molecular weight of the phosphocholine headgroup. The peak generated for each lipid class is the electronic summation of the precursor peaks which fragmented to form the neutral loss or fragment detected, i.e. a mass spectrum of the compounds. The individual phospholipids can be identified by their precursor ion mass and quantified relevant to their area or abundance. Calibration of phospholipid response factors will be performed using authentic and internal standards, quality controlled by Avanti. The fatty acid content of each phospholipid class can be calculated based on the molar ratio of fatty acid per mole of phospholipid species measured [154]. Summation of serum PC, SM and LPC will be included in the choline pool balance. The PC/PE ratio will also be derived from this quantitative MS/MS technique.

GC/FID will be used to identify and measure fatty acid species present in total lipid extracts of serum samples. The acid labile esters and free fatty acids present will be transesterified or esterified, respectively, with an acidic methanol solution to yield the corresponding fatty acid methyl esters. This procedure will yield the total fatty acid profile; to include free fatty acid and the fatty acids derived from the lipid parent structures. Calculations of n-6 / n-3 (18:2n6 / 18:3n3) ratio as well as monitoring of triene/tetraene (20:3n9 / 20:4n6) ratios will be derived from this data.

Serum and stool samples from subjects with CF will be collected at all protocol visits (baseline, 3, 6, 12, and 18 months) and analyzed as described above. With our proposed methods, each sample will provide a wealth of information; Figure 4 illustrates this point. The addition of an internal standard mixture of non-endogenous yet structurally related compounds to each sample prior to extraction will normalize recovery efficiencies and minimize reported mass, matrix, concentration and solution related ionization effects [155-157]. The proposed mixture will contain: Choline - [N,N,N-trimethyl-d_9] bromide, betaine - [*N,N,N*-trimethyl-d_9]-HCl, phosphocholine - [*N,N,N*-trimethyl-d_9] chloride, glycerophosphocholine - [*N,N,N*-trimethyl-d_9], 17:0 – 20:0 PE, 17:0 – 20:0 PC, 23:0 SM and 17:0 LPC

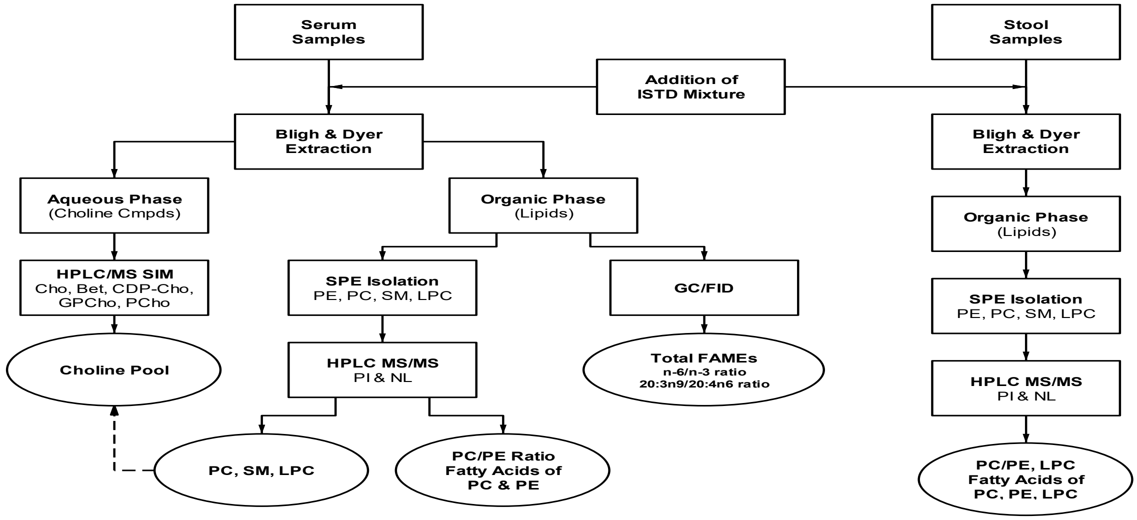

Figure 4: Flow chart (above) of proposed analytical methodology. Abbreviation: Internal standard (ISTD), choline (Cho), betaine (Bet), cytidine diphosphocholine (CDP-Cho), glycerophosphatidylcholine (GPCho), phosphocholine (PCho). PC, PE, SM, LPC, precursor ion monitoring (PI), neutral loss monitoring (NL), selected ion monitoring (SIM), fatty acid methyl esters (FAMEs), solid phase extraction (SPE), gas chromatography/flame ionization detection (GC/FID), EFA profile (n-6 / n-3), arachidonic acid trafficking (20:4 metab./anab.). Blocks represent procedures and assays. Ovals represent data generated from procedures and assays. Dotted line denotes addition of HPLC MS/MS PC, SM and LPC results to aqueous derived choline pool for summation of total.

b. Laboratory tests: A series of laboratory tests will be done at baseline, 3, 6, 12 and 18 months. Close consultation with CHOP laboratory medicine faculty, consulting and collaborating laboratories were undertaken to carefully review the laboratory testing blood requirements proposed for this study. After accounting for and consolidating complementary blood tests, as well as giving consideration to special specimen collection and processing requirements, the ideal blood volume and minimal specimen back-up sample volumes were determined based on the weight of the subject. Study subjects > 25 kg weight will have a maximum of 75 ml drawn and children less than 25 kg will have a maximum of 3 ml/kg of blood drawn. (Approximately equivalent to no more than 5 tablespoons) The amount of blood collected will be strictly based upon the weight of the child (ie: a child weighing 22 kg will have only 66 ml of blood drawn).

Complete blood count with differential will be determined using standard clinical hematologic methods. A complete metabolic panel (sodium, potassium, chloride, bicarbonate, phosphorus, glucose, calcium, alkaline phosphatase, ALT, AST, total protein, albumin, bilirubin, blood urea, nitrogen, creatinine), and GGT will be determined using standard methods. Urine will be collected at all protocol visits for a standard urinalysis. At baseline, genotype will be obtained from the medical record when available. When unknown, a blood sample will be drawn and submitted for genotype analysis (Genzyme Genetics, Pittsburgh, PA). For the purposes of this study, subjects were categorized as ΔF508/ΔF508 homozygous, ΔF508/other mutation heterozygous, and other/other genotypes. Homocysteine, methionine, cysteine, B_6, B_{12}, RBC folate, and acylcarnitine will be assessed at all protocol visits (baseline, 3, 6, 12, and 18 months) by standard methods in the CHOP Clinical Laboratory. A small amount of additional whole blood will be drawn from subjects remaining in the study after 2/1/11. High vitamin B_{12} levels have been detected in subject lab results. This can be related to elevated levels of cyanide in the blood, as the source of vitamin B_{12} in CF specific and generic vitamin supplements is cyanocobalamin. This will be analyzed by ARUP Laboratories, Inc. (ARUP, Salt Lake City, UT).

c. Fat Soluble Vitamins and Lipid Profile: Serum vitamin A (retinol), retinol binding protein (RBP), retinyl esters and transthyretin (prealbumin) for vitamin A status assessment will be analyzed at Craft Technologies Laboratories (Wilson, NC), and Vitamin E (alpha-tocopherol) will be analyzed at ARUP Laboratories, Inc. (Salt Lake City, UT). The vitamin A specimen will be protected from light. Vitamin D (25-OHD) will be determined using a radio-iodinated tracer (Dr. Bruce Hollis, Medical University of South Carolina, [158, 159]). Vitamin K status

will be assessed by PIVKA II using a kit at the GCRC at CHOP, and by determination of percent undercarboxylated osteocalcin as a fraction of total serum osteocalcin, which will be sent to Yale School of Medicine (Dr. Caren Gundberg). A lipid panel including total cholesterol, low density and high density lipoproteins will be done by standard methods at the CHOP Clinical Laboratory.
Plasma will be analyzed for plasma sterols to test the hypothesis that altered levels of these lipids occur in subjects with cystic fibrosis, and that the LYM-X-SORB™ intervention brings them closer to typical concentrations. Dr. David Russell, UT Southwestern Medical Center (Dallas, TX) developed a sensitive isotope-dilution mass spectrometry-based method to quantify 40 sterols in small volumes of plasma, iincluding two abundant sterols (cholesterol and lathosterol) and less abundant asterols (desmosterol, lanosterol, campesterol, sitosterol, cholestanol, 24-dihydrolanosterol and others). We will use available stored plasma specimens for this analysis to be run at Dr. Russell's lab[160].

d. Markers of Bone Turnover

The following markers of bone formation and resorption will be assessed at baseline, 3 month, 12 month and 18 months.
(1) Vitamin D - 25(OH)D Despite its traditional name vitamin D is not a true vitamin but rather a steroid hormone. Vitamin D can either be synthesized by the body when exposed to sunlight or it can be obtained through the diet. The major biological function of vitamin D is to maintain normal blood levels of calcium and phosphorus [161]. Serum concentrations of 25-hydroxyvitamin D is 25(OH)D are considered to be the most reliable measure of overall vitamin D status since this metabolite is the major circulating metabolite of vitamin D and it is not tightly regulated. Concentrations of 25(OH)D will be measured using a ^{125}I radioimmunoassay (Diasorin, Stillwater, MN).
(2) Osteocalcin Osteocalcin, also known as bone Gla protein, is a vitamin K-dependent, calcium-binding protein which is a major component of the noncollagenous bone matrix [162, 163]. Osteocalcin is synthesized by osteoblasts in bone [162]. During bone formation, newly synthesized osteocalcin is incorporated into the extracellular bone matrix and a small fraction is released into circulation. Because serum osteocalcin originates exclusively from bone, serum osteocalcin levels reflect bone formation activity [162]. Osteocalcin is also released into serum from the bone matrix during bone resorption. However, this circulates as non-immunoreactive fragments that are rapidly metabolized in the kidney and, to a lesser extent, in the liver [164]. Serum osteocalcin is elevated in disease states characterized by increased bone turnover such as osteoporosis and hyperparathyroidism, and low in conditions associated with low bone turnover such as hypoparathyroidism and growth hormone deficiency [162]. Serum osteocalcin measurements are also useful in osteoporosis research, and have shown good correlation with bone histomorphometric measurements [165]. Osteocalcin will be measured using a competitive immunoassay (Quidel, San Diego, CA)
(3) Intact Parathyroid hormone (iPTH) The parathyroid hormone (PTH) is an 84 amino acid peptide produced by the parathyroid gland [166]. Secretion of PTH is regulated by the level of calcium in the blood. PTH increases the calcium and phosphorus release from bone, decreases the loss of calcium and increases the loss of phosphorus in the urine and increases the activation of 25-hydroxy vitamin D to 1,25-dihydroxy vitamin D in the kidneys. Intact parathyroid hormone will be measured using an immunometric assay (Nichols Institute Diagnostic, San Juan Capistrano, CA).
(4) Fractionated Alkaline Phosphatase: From a fractionated alkaline phosphatase, we will determine bone specific alkaline phosphatase as well as liver-specific alkaline phosphatase. The skeletal, or bone-specific, isoform of alkaline phosphatase is a tetrameric glycoprotein found on the surface of osteoblast cells [167]. The function of bone-specific alkaline phosphatase (BSAP) is not clearly understood, although it's been shown to be a biochemical indicator of bone turnover [168]. From this test we will also assess liver alkaline phosphatase as a measure of liver function. Serum samples will be sent to ARUP Laboratories, Inc. (Salt Lake City, UTAH) for analysis.

e. Stool Collection and Analysis: At screening, random stool samples will be obtained for assessment of fecal elastase 1 to determine the level of pancreatic enzyme activity, and to confirm pancreatic status. Subjects will be provided with the stool collection kit and proper instructions and supplies, and will bring a stool sample back at their next study visit or send their sample directly to the lab for processing. The stool sample will be stored at -20°C, and analyzed with an enzyme-linked immunosorbent assay kit sent to Genova Diagnostics Laboratory

(Asheville, NC). For purposes of this study, only subjects with evidence of minimal pancreatic lipase activity (fecal elastase <15 μg/g stool) will be enrolled. Subjects will perform 72-hour stool collections at baseline, 3 and 12 month visits. These stool collections will be performed at home and sent to CHOP on dry ice. Subjects will be given a home collection kit and detailed instructions. Specimens will be stored frozen until analysis of total fat content by a gravimetric method (Mayo Medical Laboratories, Rochester, MN). 3-day weighed food records will be collected to coincide with the 72-hour stool collection and total dietary intake of fat during the 3-day period will be assessed (see below for dietary assessment methods), and the coefficient of fat absorption (%CoA) will be calculated [141]. An aliquot of homogenized frozen stool will be sent to Avanti Polar Lipids, Inc. for stool phospholipids analysis.
For the description of the microbial population in stool, an aliquot of frozen stool will be sent to Dr. Frederick D. Bushman's lab for fecal microbiome analysis (Bushman Lab, Perelman School of Medicine, University of Pennsylvania, Philadelphia, PA). We will analyze available stored stool samples from baseline before supplementation and from the 3 and/or 12 month stool sample collected after supplementation with LYM-X-SORB™ or placebo.

f. Metabolic, Inflammatory, and Infection Assessments:

(1) Pulmonary function: Pulmonary disease will be evaluated at CHOP at baseline, 3, 12 and 18 months using standard methods for spirometry [169, 170]. Forced expiratory volume at 1 second (FEV_1), the primary pulmonary outcome, will be measured and expressed as a percent of the reference values [171]. Maximum inspiratory pressure (MIP) and maximum expiratory pressure (MEP) will be measured to assess respiratory muscle strength using standard techniques [97, 98, 172]. At baseline and also at the 18-month visits, a sputum sample will be obtained from subjects for assessment of microorganism colonization of the lungs. In the interpretation of FEV1 as an outcome measure of pulmonary function, it is important to take lung infection into account as a confounding factor. Sputum cultures will be sent to the Microbiology Laboratory and CHOP for analysis.

(2) Bile acids in urine and serum will be analyzed at baseline and 12 months using fast atom bombardment ionization mass spectrometry, the standard technique. Bile acids and bile acid conjugate compounds are detected, and patterns of bile acid abnormalities are identified. Interpretation by trained personnel is important. We will send our samples to the Clinical Mass Spectrometry Laboratory at the Children's Hospital Medical Center in Cincinnati, OH (Kenneth DR Setchell, PhD, Director).

(3) Magnetic Resonance Imaging and spectroscopy (MRI/MRS, Siemens Advanto 1.5T) without contrast, to assess the subjects for fatty liver at baseline on a subset of 24 subjects, 10 years of age or older who are able to cooperate with the procedure without sedation. A follow-up MRI/MRS will be performed to document changes in fatty liver status after 12 months of supplementation with LYM-X-SORB™ or placebo powder. Approximately 50 of the subjects will be 10 years of age or older, and 24 subjects will be chosen, with similar distribution by sex and age. Non-invasive MRI of the liver detects the presence of steatosis. Subjects will undergo a MR scan to quantify the amount of fat in the liver and to estimate the relative amounts of visceral and peripheral fat. The subject will be positioned supine on the MR scanner bed. Small plastic containers filled with fat of a known concentration may be placed on the subjects anterior abdominal wall to serve as reference standards for the fat quantification analysis. A standard MR body coil will be placed over the patient's abdomen. Both MR imaging and spectroscopy sequences will be acquired. Respiratory gating may be used with some of the acquisitions. The total MR acquisition time is expected to be less than 45 minutes and the total time required for the MR scan is expected to be approximately 1 hour. MRI has been used to study steatosis and CF [44] and MR spectroscopy sequences will also be acquired to quantify hepatic triglyceride content [46-48]. The following MRI scans will be performed: coronal T1; axial T2 TSE FS, and for hepatic fat fraction, in phase and out of phase gradient echo with flip angles (70 ,,20) to resolve ambiguity of the dominant T2* correlation. MR Spectroscopy (MRS) will be performed using the single voxel method. Using a water-unsuppressed SVS the lipid content of the VOI region will be measured against the internal water reference. To minimize T2 loss, a short TE time (20 msec), a long TR time (4 sec) will be used to minimize the T1 saturation effect. A stimulated-echo technique will be used to ensure the shorter TE time. The SVS voxel will be placed in the same location each time to avoid possible heterogenicity of lipid distribution in the liver.

Single-voxel (2x2x2 cm) MRS (Siemens 1.5T Avanto) will be used to determine lipid and choline contents of the lower leg muscle. Similar to the liver studies, single voxel localization with STEAM technique with a TR of 4 sec and TE of 20 msec will be utilized with 40 acquisitions. The total acquisition time for this MRS is 2.5 minutes. Additional MR sequences may be preformed to optimize the fat quantification protocol.

(4) Inflammatory cytokines will be measured as markers of inflammatory status using serum levels of high sensitivity C-reactive protein (HS-CRP). These will be assessed for each protocol visit (baseline, 3, 6,12, and 18 month). HS-CRP will be used as a marker of the acute phase response to inflammation and infection. Serum samples will be sent to ARUP Laboratories, Inc. (Salt Lake City, UT) for analysis of HS-CRP.
g. Resting Energy Expenditure: Using indirect calorimetry, resting energy expenditure (REE) and respiratory quotient (RQ) will be assessed at baseline, 3, and 12 months in the early morning after an overnight stay in the inpatient GCRC unit at CHOP. REE will be measured by open circuit indirect calorimetry using a computerized metabolic cart (SensorMedics SPECTRA or ENCORE, Yorba Linda, CA). In preparation for the REE, each subject receives a standardized evening meal and fasts from food and medication for 12 hours before the REE measurement. In the early morning the subject is awakened and restricted to minimal physical activity. A 60-minute REE test will be performed between 7:00 and 10:00 AM with the subject resting quietly under a clear, plastic hood watching a videotape. Data from the first ten minutes and during periods of significant physical movement or coughing are eliminated, with the remaining data averaged for the mean REE and RQ. REE is calculated from the modified Weir equation [173], using oxygen consumption and carbon dioxide production. REE is subsequently compared to predicted values derived from the World Health Organization that adjust for age, gender and weight [174] and Schofield equations that adjust for age, gender, weight and height [175], and for fat-free mass.

h. Dietary Intake: Dietary intake will be assessed at baseline, 3, 12 and 18 months using 3-day weighed food records. Subjects and parents/guardians will be asked to weigh and record the kinds and amounts of all foods and beverages consumed during this 3-day period. Families will be provided with measuring cups, spoons and an electrical digital food scale to use at home for the 3-day weighed food record. Detailed verbal and written instructions will be provided to ensure that the recording procedures are clearly understood. Completed diet records will be reviewed and analyzed by skilled research bionutritionists. The Nutrition Data System (NDS) program (University of Minnesota Nutrition Coordinating Center, School of Public Health) [176] database contains over 16,000 food items, and is continually updated to reflect changes in the marketplace. Dietary reference intakes (DRIs) for energy, protein, EFA (both n-6 and n-3 fatty acids), and choline have been recently updated [27, 177]. To assess the adequacy of intake of EFAs, we will use these new DRIs to compute percent of recommended intake of n-6 fatty acids (linoleic acid as indicator) and n-3 fatty acids (α-linolenic acid used as indicator).

i. Anthropometric Assessment: All anthropometric techniques will follow those described by Lohman et al [178]. Weight (0.1 kg) will be measured on a digital electronic scale (Seca, Munich, Germany) and stature (0.1 cm) on a stadiometer (Holtain, Crymych, UK. Skinfold thickness will be measured (0.1 mm) at the triceps, biceps, subscapular, and supra-iliac sites with a skinfold caliper (Holtain, Crymych, UK) to assess subcutaneous fat stores. Circumferences will be measured at the mid-upper arm, and maximum calf measured with a non-stretchable fiberglass tape (0.1 cm) (McCoy, Maryland Heights, MO). All measurements will be taken and recorded in triplicate and the mean used in analyses. Measured (reported when necessary) heights of biological parents will also be recorded to estimate genetic growth potential [179]. The Research Technician will be fully trained in anthropometric techniques by Dr. Schall and will conduct anthropometric evaluations at each visit. Anthropometric equipment is checked and calibrated before every assessment to assure proper operation.

j. Body Composition: Body composition, total fat-free mass and fat mass and percent body fat, will be assessed by the four skinfolds described above using prediction equations adapted for children and adolescents [180, 181], and also using dual energy x-ray absorptiometry (DXA). Whole body scans (Delphi A, Hologic, Inc., Bedford, MA) will be done at baseline, 3, 12 and 18 months to assess body composition including total fat-free mass, fat mass and percent body fat. The DXA methodology uses very low-dose x-ray exposures (3 mrem) and measurements are rapid, making this a suitable technique for use in children. The measurement

takes approximately five minutes. Subjects will be scanned in hospital scrubs to assure consistency from visit to visit, and eliminate artifacts of attire such as snaps and buttons. Standard positioning techniques are used. The scans will be analyzed using standard analysis protocol, and stored on super disks. Any interfering factors that are not removable, such as gastrostomy tubes, metal implants and belly button rings are noted on the data collection form. A urine pregnancy test will be performed on female subjects who have had a menstrual period or who are ages 9 and older prior to the scan. Pregnant subjects will not be scanned and will leave the study protocol. Quality control scans are performed daily using a simulated L1-L4 lumbar spine made of hydroxyapatite encased in epoxy resin. Monthly printouts of quality control scans will be reviewed. Quality control assessments will also be done using a whole body composition phantom. A difference of >1.5% from the standard will be deemed out-of-range requiring servicing by the manufacturer.

k. Bone Health Measures - Whole body and Spine DXA for bone mineral content and bone density, and the pQCT for bone density and structure. Whole body DXA will be done at Baseline, 3 month, 12 month and 18 month visits. Spine DXA and pQCT will be done at Baseline, 12 months and 18 months visits.

(1) Dual Energy X-Ray Absorptiometry (DXA): Bone mineralization of the anterior-posterior spine and whole body will be assessed by DXA using a Hologic Delphi A bone densitometer (Hologic, Bedford, MA) in all subjects. The DXA methodology uses very low-dose x-ray exposures (3 mrem) and measurements are rapid, (5 minutes/scan) making this a suitable research and clinical technique for use in children. See above for more detailed description for DXA. The scans will be analyzed to generate traditional DXA measures: vertebral Area, BMC and areal-BMD (gm/cm^2), and whole body Area, BMC and areal-BMD (gm/cm^2). Long-term reproducibility is calculated within the QC subroutine of the manufacturer's software. Phantom scans are stored on super disks daily and back-up storage of the QC database is conducted weekly. In our institution, the in vitro CV is less than 0.6% and the in vivo coefficient of variation in adults is less than 1%. The calibration differences between instruments from the same manufacturer of less than 1% [182].

(2) pQCT Measures of Bone Dimensions, Density and Strength and Muscle Dimensions:
pQCT measures will be performed at all protocol visits using the Stratec XCT 2000 peripheral quantitative computer tomography scanner (Orthometrix, White Plains, NY). We will measure both radius and tibia. The impact of impaired biomechanical stimulation and decreased modeling will be more pronounced at the tibia, a weight-bearing site. Cortical thickness is greater in the tibia compared with the radius, and therefore will be less subject to partial volume effects. The XCT-2000 is a rotate-translate computerized tomography device with a 12 detector unit using a slice thickness of 2.3 mm. The standard voxel size is 0.4 mm^2 and the scan speed 25 mm/sec. The scans require approximately five minutes at each site and involve minimal radiation exposure. Measurements will be taken at the tibia ultradistal site (3% length of the tibia from the distal endplate), which is predominantly trabecular, and at the cortical 38% site (see Figure 5). The 38% site was selected since this is the site of maximal cortical thickness, to avoid partial volume effects. After a scout scan to identify the tibia endplate, the CT cross-section is then automatically set at 3% of the tibia length proximal to the identification line for the determination of trabecular BMD (gm/cm^3) and trabecular cross-sectional area (mm^2) and at the 38% site for determination of total cross-sectional area (mm^2), cortical cross-sectional area (mm^2), cortical thickness (mm), and cortical density (gm/cm^3). For the radius, measurements will be taken at 2 sites, the 3% (predominantly trabecular) and 30% length (predominantly cortical) of the radius from the distal endplate forearm length will be measured to determine these sites. One additional site on the tibia, 66% the length of the tibia from the distal endplate, will be scanned. This is an ideal

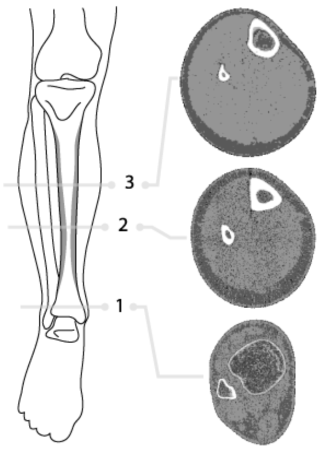

Figure 5. pQCT Sites.
The figure shows tibia measurement sites and their corresponding pQCT cross-sections.
Site 1: 3% from the endplate reference point, ideal for analysis of trabecular properties.
Site 2: 38% of the tibia length from the distal endplate, notable for maximum cortical thickness, ideal for cortical bone analyses.
Site 3: 66% of the tibia length from the distal endplate, notable for maximum muscular cross-sectional area, ideal for soft tissue analyses.

site for soft tissue analysis of the lower leg and notable for maximum muscular cross-sectional area. Precise and accurate determination of bone measures using pQCT and DXA requires a calibration standard to compensate for and reduce the effects of beam-hardening artifacts and scanner drift [183]. Quality control (QC) is monitored using the manufacturer-provided phantom composed of hydroxyapetite encased in acrylic. Cone phantoms are scanned daily and measurements are automatically archived in the QC database with back-ups on compact disks conducted weekly. The Stratec pQCT provides a high degree of precision and accuracy [184, 185]. Short-term precision in vivo, expressed as root mean square standard deviation (RMSSD) of paired measurements of 20 healthy volunteers was 0.5%; structural parameters, such as the cross-sectional moment of inertia showed an excellent short-term precision at the distal femur, with RMSSD = 1.2 % [185].

The calculation of bone stress-strength index is derived from the pQCT measures of bone density and dimensions as follows: SSI (mm^3) = $\Sigma\ [[(a_i \times d_i^2)(CD_i/ND)]/d_{max}]$. The CD is the cortical density within a given voxel i, ND is normal cortical density (1200 mg/cm^3), a_i is the area of a given voxel i, d_i is the distance of voxel i from the center of gravity, and d_{max} is the maximal distance of a voxel from the center of gravity. Because the individual voxels are weighted to ND, this measure is not subject to partial volume effects. This index accurately and precisely describes the mechanical properties at the structural level [186].

I. Muscle strength: Assessments of muscle strength will be conducted at Baseline, 3 month, 12 month and 18 month visits. Three techniques for assessing muscle strength will be utilized in this study and subjects will have assessments at each protocol visit to correspond to measures of bone mass and mineralization.

(1) Biodex: muscle strength will be assessed using the Biodex Multi-Joint System 3 Pro (Biodex Medical Systems, Inc, Shirley, NY) after a 5-minute warm-up period on a treadmill. Biodex is considered the gold standard for muscle strength assessment. High intrarater (0.97 to 0.99) and interrater (0.93 to 0.96) intraclass correlation coefficients have been reported [187]. Muscle efficiency will be calculated as muscle force/muscle cross-sectional area. Maximal isometric plantar flexion and dorsiflexion of the left ankle will be tested*as well as the left quadriceps (thigh muscle).* Each subject will go through a period of standardization and familiarization prior to testing. The subjects will be seated in an upright position (hip angle 90˚-100˚) with their back tight against the testing chair, which is firmly attached to the dynamometer. The knee joint will be stabilized at 0-10˚ flexion. The foot will be placed on the foot plate of the dynamometer such that the anatomical axis of rotation of the ankle joint and the axis of the dynamometer are aligned. The neutral position (0˚) will be set at a 90˚ angle between the foot and the tibia. The body will be restrained with straps at the level of the foot, calf, thigh, pelvis and chest. Maximal isometric plantarflexor strength will be tested with the ankle at the defined 0˚ neutral position and at 10˚ plantar flexion [188]. Maximal isometric dorsiflexor strength will be tested with the ankle at 20 and 30˚ plantar flexors and the left quadriceps *maximum isometric strength (lateral knee extension and flexion) will be tested at the 60°/s angular velocity*. The subjects are instructed to pull on the footplate and the highest torque of 5 contractions (5 sec each with 60 sec intervals) is recorded. A biofeedback system will be used to ensure that all subjects perform contractions with maximal effort.

(2) Force Plate: Muscle strength will also be assessed using a Force Plate (Kistler Quattro-Jump Force Plate, Kistler Instrument Corp., Amherst, NY). The Force Plate will electronically record the strength of the muscles that are used in jumping. The height of the jump, jump power, and other parameters are calculated from the take-off force and force of landing is also assessed to estimate muscle strength. After 5 minutes of treadmill walking for warm-up, each subject will perform the force plate test by completing approximately 3 practice jumps followed by 3 test jumps that will be recorded for measurement of strength. This test takes 10 minutes to complete.

(3) Hand Grip Dynamometer: Functional assessment of the upper limb strength will be obtained using a hand-grip strength dynamometer (Takei Scientific Instruments Co., Ltd., Japan). This hand held device is capable of measuring instantaneous hand strength as a function of time for periods of up to 300 seconds. The subject

stands upright with arms extended and is instructed to grip the dynamometer and exert full force. Three trials are performed using each hand. The dynamometer digitally displays the force production (kgf) by the subject.

m. Pubertal Status:

Pubertal status will be determined using a validated self-assessment questionnaire developed by Morris and Udry [189]. The self-assessment scoring system uses the Tanner Stages of sexual maturity, with pictograms and descriptions for the stages of pubic hair for both genders, genital development in males and breast development in females. Results with this questionnaire in children with Crohn's disease showed excellent agreement with physician assessment [190]. The self-assessment questionnaire will be completed by all subjects 8 years of age or older (assisted by a family member as needed). For quality assurance and accuracy, the results of the questionnaire are inspected immediately for plausibility. If the result seems implausible based on the age of the participant, we will review the questionnaire with the participant and/or parent/guardian to review the directions and re-assess the subject. Female subjects will asked a series of questions to determine their menarcheal status, in particular, age at first menstruation,

n. Adherence, Health History and Demographic Questionnaires:

(1) Adherence: Adherence will be systematically assessed on a regular basis using the following methods: 1) Supplements will be shipped to participants' homes each month and at the end of each month, families will be contacted to record the number of unused packets from the previous month. 2) Monthly calendars will be provided to participants at each visit with instructions to record each day the supplement is taken as directed. Calendars will be mailed in monthly or collected at the protocol visits . 3) A semi-structured interview using a modified nutrition module of the Medication Adherence Measure [191] will be conducted with subjects and families during each visit to determine adherence to supplementation. Attempts to maximize adherence to supplementation will include the following methods: 1) At each visit LYM-X-SORB™ or placebo powder will be provided along with written suggestions for a variety of food and beverage mixtures with proven acceptability in children and adolescents with CF in order to optimize palatability and avoid taste fatigue. 2) Participants will be told they will be awarded 1 point for each day of supplement consumption as directed with no additional points rewarded for extra supplementation in a day. Points will be determined by project staff utilizing both objective and subjective data described above and will be traded in for a gift card in the dollar amount that corresponds to the number of points each participant earned. The child will have the choice of gift card vendors (e.g., cinema, DVD/video game rental store, shopping mall). This will recur each month for each participant. Parents of children under age 12 will be encouraged to place stickers on the aforementioned calendars to indicate supplement consumption as this method has been shown to increase motivation in younger children who have more difficulty assessing long-term benefits of adherence. 3) Finally, we will maintain regular contact with participants via telephone to ensure delivery of monthly supplement supply and obtain a cursory assessment of barriers to supplement adherence so that we can develop individualized strategies to overcome these barriers. We expect that this phone call will last about 10 minutes. We will also send birthday and holiday cards to all subjects in the study.

(2) Disease Severity Assessment: In addition to pulmonary function testing, we will assess disease activity using days of IV antibiotic use, unscheduled (sick) patient visits, hospital and emergency room visits as indicators. We will gather this information from the calendars and interview at each visit and then verify the information from the medical records. We will document admissions to the hospital for the year prior to participation in the study and then prospectively for the 18 months of the study. We will work with the clinical care team at each of the CF Centers to capture both CF-related illness and non-CF-related illnesses. Discharge diagnoses, complications, and Emergency Department visit information will be collected. Results for selected diagnostic tests (i.e., liver, microbiology, virology) will be collected.

(3) Adverse Events Diary: Subjects will keep a home diary of all adverse events, and rate by intensity (mild, moderate, severe). These will be collected at each visit and a new diary supplied for the next 3-month period. Serious adverse events will be reported as per various policies in a timely manner to the NIH, CHOP IRB, GCRC, Study PI, CF Center, and Avanti.

(4) Family and Health Questionnaire: The questionnaire will be administered by interview by the research staff, and will consist of two sections. The Health History and Diet section has general questions about the subject's health history including documentation of medical history, recent hospital admissions and illnesses, medication, pancreatic enzyme supplementation, chest physiotherapy, and nutrient supplement use. A Fracture History Questionnaire will be administered at the baseline visit to establish the history of all bone fractures that the subject has sustained, and a Fracture History Update will be administered at the 18 month visit to determine if any fractures were sustained during the course of the study. The Family Environment section describes aspects of environment, such as parental education and income level, and household size to describe the demographic characteristics. In addition to family contact information (name, address, phone numbers), contact information from two non-household contacts will be collected to maintain contact with the subject in the event that the subject cannot be contacted at their primary residence.

(5) Quality of Life (QOL) Questionnaires: All subjects will be administered 2 QOL measurements: a pediatric questionnaire, the PedsQL 4.0 [192] and a CF-specific questionnaire (CF-QOL) which is adapted for preadolescent children [193] and for adolescents and adults [101]. The PedsQL is a modular instrument designed for measuring HRQOL in children aged 2 to 18 years of age. The PedsQL 4.0 Generic Core Scales are a multidimensial child self report and parent proxy report scale developed as a generic core measure to be integrated with PedsQL Disease-Specific Modules. The Generic Core Scales (physical, emotional social and school) consist of 23 items applicable for healthy school and community populations with acute and chronic health conditions [192] and have been validated in a number of studies for various disease states. These measurements will be performed at baseline, 6, 12 and 18 months by trained research coordinators and will take approximately 20 minutes to complete. Dr Kevin Hommel who is an expert in these measurements will interpret and analyse the results of de-identified information.

o. Biostatistical Design and Analysis

(1) Sample Size Considerations: This is a double-blind, placebo-controlled, randomized 1:1 controlled trial to evaluate the effectiveness of the next generation LYM-X-SORB™ powder on the fatty acid and choline status among children with CF and PI. There are three primary outcome measures (linoleic acid, triene/tetraene ratio, and the PC/PE ratio). We propose that after 12 months of supplementation with LYM-X-SORB™, subjects will show improved EFA status as indicated by an increase in serum linoleic acid (18:2n-6) and a decrease in the triene/tetraene ratio (20:3n9/20:4n-6 ratio), and improved choline status as indicated by an increase in the PC/PE ratio when compared to patients on placebo. The sample size estimation was based on a t-test of values at the 12 months visit. Because there are three outcome measures, the Tukey, Ciminera, and Heyse [194, 195], correction for multiple comparisons was used and the alpha level was set to 0.0292. Data from a study by Lepage et al [7] show that children with CF have linoleic acid levels (mol%) of 17.9 (SD 1.9) and 16.7 (SD 2.6) while children without CF have linoleic acid level of 19.8 (SD 2.2). The triene/tetraene ratio in children with CF is .041 (SD .02) and .037 (.02) and in children without CF is .012 (SD .0004). Data from a study by Innis et al [4] indicate that the PC/PE ratio in children with CF is 10.9 (SD 10.9) and in children without CF is 29.7 (SD 16.5). We used the maximum variability observed and the minimum clinically significant difference for all sample size calculations. A sample size of 64 subjects will have 80% power (alpha=0.0292, two sided) to detect a mean difference of 2 mol% in serum linoleic acid when the standard deviation is 2.6. This sample size will also provide more than 80% power to detect a mean difference of .03 in the triene/tetraene ratio and a mean difference of 20 in the PC/PE ratio. We will enroll a total of 112 subjects to account for a 15% expected attrition rate.

(a) Randomization and Stratification: To achieve a balance LYM-X-SORB™ and placebo regarding gender and age, subjects will be first assigned to one of four strata on the basis of gender and two age groups (5.0 to 11.9 and 12.0 to 18.9 years). Within each stratum, subjects will be randomly assigned to the treatment or to the control group. This will ensure an equal representation of younger and older children and boys and girls in each treatment arm.

(2) Data Analysis: Analysis will begin with descriptive analyses of the study sample using means, standard deviations, medians, and ranges for continuous variables, and frequency distributions for categorical variables. Descriptive statistics and exploratory graphing such as frequencies, means, standard deviations, box plots, and scatter plots will be used to assess the normality of the data in terms of the presence of skew and/or outliers. The association between continuous variables will be measured by Pearson's correlation coefficient or Spearman's rho coefficients as appropriate. Prior to testing hypotheses, baseline demographic and clinical characteristics will be compared between the two treatment groups: placebo versus LYM-X-SORB™ . The purpose of these comparisons is to examine whether any adjustments to overall group comparisons on the outcome variables will be necessary. These statistical comparisons will be performed by chi-square tests for dichotomous or nominal variables, by t-tests for continuous variables, and by Wilcoxon rank-sum tests for non-normally distributed continuous variables. All three primary outcome measures (linoleic acid, triene/tetraene ratio, PC/PE ratio) will be examined for normality. If any continuous outcome measure is skewed, appropriate normalizing transformation will be applied prior to the primary analyses.

(a) Primary Aim: The primary aim examines the long-term effect of LYM-X-SORB™; the focus of the primary aim are EFA and choline status measured 12 months following the baseline visit via two hypotheses. ***Hypothesis 1*** postulates that LYM-X-SORB™ will improve the EFA status (increase linoleic acid and decrease the triene/tetraene ratio) compared to placebo in subjects with CF and PI. ***Hypothesis 2*** postulates that LYM-X-SORB™ will improve choline status (increase the PC/PE ratio) compared to placebo in subjects with CF and PI. The expectation is that EFA and choline levels for subjects receiving LYM-X-SORB™ will approach those levels found in healthy people. Thus, in order to investigate Hypothesis 1 and 2, we will use summary data on EFA and choline levels in healthy subjects from published literature. The levels in healthy subjects were also used to calculate the sample sizes above. To investigate these hypotheses, we will analyze each of the three outcomes separately and use the cross-sectional outcome data obtained at the 12-month visit.

The primary analyses will be carried out via t-tests to test the hypothesis that the means are not equal among subjects with CF on placebo and subjects with CF on LYM-X-SORB™. Since this is a clinical trial with three primary outcome variables, a Type I error of .0292 will be specified. If analyses indicate demographic and clinical measures (including the outcome variables at baseline) that are substantially different between the two groups, those measures will be included as covariates, and regression based Analyses of Covariance (ANCOVA) will be performed. The dependent variable in the ANCOVA is the outcome at the 12-month visit, treatment group (placebo vs. LYM-X-SORB™) is the qualitative factor, the demographic, clinical measures and the outcome level at baseline, the covariates. These models will be fit using SAS procedure PROC GLM. The means for each outcome for LYM-X-SORB™ group will be calculated for their inclusion in the 95% confidence interval for normal values obtained from published studies. For example, the mean linoleic acid level for subjects taking the current generation of LYM-X-SORB™ has reached a normal healthy level if it lies in the published 95% confidence interval for normal values.

Secondary analyses. Since each primary outcome is measured at five time points over the study period, secondary analyses will be performed using all interim measures. A mixed-effects model that accounts for multiple measurements per study subject will be applied [196]. Specifically, each outcome variable will be regressed on an indicator variable that identifies the treatment group, time of each study visit, and other covariates found significant in the demographic comparisons described above. Mixed-effects models allow the existence of missing data due to attrition. Plots of the data over the study period for each treatment groups will be examined for curvilinear patterns and a quadratic term will be added accordingly. Parameter estimates can be obtained by maximizing the log likelihood function. Models will be fit using SAS procedures PROC MIXED and PROC GENMOD. Secondary analyses will be done at a Type I error of .05.

(b) Secondary Aims: ***Hypothesis 3:*** In subjects with CF, those receiving 12 months of LYM-X-SORB™ supplementation will show improvements in essential fatty acid status (serum fatty acid profile), choline status (serum LPC, homocysteine, methionine, vitamins B_6, B_{12}, acyl carnitine, and RBC folate, fecal PC), fat soluble vitamin status, bile acid composition, inflammatory cytokines, resting energy expenditure and respiratory quotient, and the incidence of fatty liver on a subset of 24 subjects, when compared to those receiving placebo. We will analyze each of the outcomes separately and use the cross-sectional outcome data obtained at baseline and the 12-month visit. Analyses will be carried out by using analysis of covariance (ANCOVA). The

dependent variable in the ANCOVA is the outcome at the 12-month visit, treatment group (placebo vs. LYM-X-SORB™) is the qualitative factor, and the outcome level at baseline, the covariate. Since this is a secondary aim, a Type I error of 0.05 will be specified. If analyses indicate demographic and clinical measures that are substantially different between the two treatment groups, those measures will be included as covariates. These models will be fit using the SAS procedure PROC GLM. ***Hypothesis 4:*** In subjects with CF, those receiving 18 months of LYM-X-SORB™ supplementation will show improvements in growth, body composition, pulmonary status, and bone health when compared to those receiving placebo. We will analyze each of the outcomes separately and use the cross-sectional outcome data obtained at baseline and the 18-month visit. Analyses will be carried out by using ANCOVA. The dependent variable in the ANCOVA is the outcome at the 18-month Visit, treatment group (placebo vs. on LYM-X-SORB™) is the qualitative factor, and the outcome level at baseline, the covariate. Since this is a secondary aim, a Type I error of 0.05 will be specified. If analyses indicate demographic and clinical measures that are substantially different between the two treatment groups, those measures will be included as covariates. These models will be fit using the SAS procedure PROC GLM. Since each of the outcomes variables is measured at 3 to 5 time points over the study period exploratory analyses using mixed-effects models will be performed by using all interim outcome measures. Specifically, each secondary outcome variable will be regressed on an indicator variable that identifies the treatment group, time of each study visit, treatment by time interaction, and other covariates found significant in the demographic comparisons described above. Plots of the observed data over the study period for each of the two treatment groups will be examined for curvilinear patterns and a quadratic term will be added accordingly. For continuous outcomes, parameter estimates can be obtained by maximizing the log likelihood function. For binary or count outcomes, parameter estimates can be obtained by solving the generalized estimating equations (GEE). These models will be fit using the SAS procedures PROC MIXED and PROC GENMOD. These analyses will be done at a Type I error of .05.

(c) Adherence

We will calculate an adherence score at each study visit using each method of assessment. This score will be the amount of powder presumed taken by subjects, expressed as a percentage of the amount of powder provided. We will compare adherence between placebo and active treatment groups via a mixed-effect models. Specifically, each adherence score will be regressed on an indicator variable identifying the treatment group, time of each study visit, and a time by treatment interaction. In addition, we will calculate the percent of adherence with 95% CI for each method.

p. Data Management: We will establish a computerized database to store study data. The design of the database will utilize standard software (e.g. FileMakerPro or Access) and will include all information collected in the study. The database will be designed to perform automatic computations, such as exact age based upon birth date and date of exam, and averaging anthropometric measures, which are recorded in triplicate. The database will allow both easy creation of data collection forms and data entry using computer screens that are identical to the data collection forms. Raw data and standardized scores obtained from all data collection points will be entered into the database and checked using procedures for double verification and range checks. Through queries of the database, we will create summary reports as needed, and prepare data sets for statistical analyses. Reports containing the number of children enrolled and data entered for each subject are generated and reviewed each month by the principal investigator. Subsets of the data will be converted into suitable format (STATA or SAS) for analyses.

All subjects will be assigned a unique identification number that will be used to insure strict confidentiality. The databases will be password protected to insure confidentiality and security. For data backup, the research team will utilize a variety of safeguards to protect the study from loss of data. Routine backup of all study files, including images of forms, the main study database, files created for analyses, and programs used to process and analyze data will be completed. The main study database will be archived on a daily basis. Other files and programs will be fully archived once every week with an incremental back-up of new or changed files every night. Every two weeks, the backup will be stored off site.

q. Data Safety and Monitoring Plan: The CF Foundation is supporting this study by providing the Data Safety and Monitoring Board (DSMB) for this study. See Human Subjects section for a more detailed description of the composition and activities of the DSMB. Serious adverse events will be monitored in real time by the

DSMB throughout the study. As allowable by the CHOP IRB, we will exclude hospitalizations secondary to exacerbation of cystic fibrosis from serious adverse event reporting. SAEs will be reported by the Principal Investigators to the Therapeutic Development Network Coordinating Center of the CFF within 24 hours of learning of the event. SAEs will also be reported to the CHOP IRB, the home CF Center IRBs and the NIH. The CHOP IRB and GCRC will also monitor safety, along with the study team throughout the study.

7. Potential Difficulties, Limitations and Hazards

The CHOP research team has extensive experience with successfully conducting CF research and nutritional intervention studies in a variety of populations. Nevertheless, the major potential difficulty of the proposed study is to recruit and retain the subjects and families and to maintain adherence to the supplementation program for an 18 months period. The most important aspects of subject retention are the ease of scheduling study visits, an organized and pleasant experience during each visit, and the financial reimbursement program. We will reimburse subjects/families at a standard rate to offset incurred expenses for travel, food, and babysitting expenses. Subjects/families will receive $100 for the baseline, 3 and 12 month visits as the overnight visits, and also at 18 months, the final visit, all of which will occur at CHOP. They will receive $50 for the 6-month visit at their own CF Center. The total reimbursement for subjects will be $450. In addition, subjects will be given an age-appropriate gift card for book/DVD/video/CD (average value = $25) at each month interval as a thank-you for participating in the study and continuing to take the supplement. The amount of the gift will be contingent upon the number of points the subject receives for taking the supplement each month (1 point per day worth $1/day if they take the supplement). We anticipate an adherence rate of ~80-85% corresponding to a monthly gift of ~$25 for each subject. It is essential that the study not add to the already significant financial burden associated with the care of a child with a chronic illness, therefore all research test costs will be covered by the research protocol and not be charged to the families' insurance payers or families. In addition, subjects will be provided coupons for the purchase of food items throughout the study for delivery of the LYM-X-SORB™ supplement.

PROTECTION OF HUMAN SUBJECTS

1. Risks to the Subjects

a. Human Subjects Involvement and Characteristics

For subjects with CF there are no exclusions for ethnic/racial background and every effort will be made to recruit children from all ethnic groups. It is well known that CF is more common in people of European ancestry, so most of our subjects will be Caucasian. Children and adolescents are a special class of subjects. The study does not involve any other special class of subjects. Seventy-eight subjects, ages 5.0 to 18.9 years, will be enrolled into this study, with a similar distribution of males and females, and a similar distribution of subjects in two age groups 5.0 to 11.9 and 12.0 to 18.9. Subjects will be recruited from ten CF Centers including CHOP. Subjects will be diagnosed as having CF with severe PI (based upon fecal elastase 1 level <15 ug/g stool (monoclonal assay). Subjects will be classified into the ethnic/racial category according to the subjects'/parents' identification preference. Based on preliminary data, the study population is expected to include approximately 94% Caucasian, 2% African-American, 0% Native American (American Indians or Alaskan natives), 4% Hispanic and 0% Asians/Pacific Islander, and 0% Other.

Subjects will be excluded if they are pregnant, have other chronic diseases and/or conditions or are taking medication that may affect gastrointestinal absorption, growth, and/or nutritional status. Subjects who become pregnant in the course of the study will not complete the study. If a positive pregnancy test result occurs during the study procedures (DXA pregnancy test or is self-reported), CHOP protocols will be initiated to involve the appropriate support personnel (e.g., social work, adolescent medicine) for informing the participant (and family, as appropriate) of the positive pregnancy test. Subjects who are taking daily vitamin and mineral supplements without EFAs or choline are eligible. Subjects taking daily EFA supplements or choline are not eligible for the Study. Subjects who are willing to discontinue taking these supplements for the duration of the study will be eligible, with the approval of subject's CF care provider, after a two-month period off the supplements.

b. Sources of Materials
Subjects will provide the research material from body measurements, questionnaires, medical records, blood, stool, urine and sputum, which will be used for analyses. Records will be kept of biological sample collections, dietary intake, and general health status. All biological samples, research records, and data obtained from this project will be used for research purposes only. Each subject will be assigned a study number and the specimens, records and data will be coded. If a result from a research test unexpectedly appears to be important for immediate clinical care, these data will be provided to Drs. Stallings, Mascarenhas and Maqbool as the physician investigators. They will discuss with the subject/family and, with their permission, provide the information to the home CF Center clinical team.

The research staff will utilize a variety of safeguards to protect the study from loss of data. The staff will perform routine backup of all study files, including images of forms, the main study database, files created for analyses, and programs used to process and analyze data. The main study database will be archived on a daily basis. Other files and programs will be fully archived once every week with an incremental back-up of new or changed files every night. Every two weeks, the backup will be stored off site. Access to the main study database is through password protection and enforcement of user privileges. Records of individuals are stored with ID numbers and a name code with no discernible personal identifiers.

c. Potential Risks
The risks for participation in the study are minimal. A study monitoring group (details in Methods Section) will oversee the conduct of the study and review progress and reports of adverse events and serious adverse events. Specific risks and minimization of these risks are described below. The risks of drawing blood are minimal, and include temporary discomfort from the needle stick, bruising and rarely an infection at the site. Each subject will have no more than approximately 5Tbsp. of blood drawn at each study visit. Experienced GCRC pediatric research nurses and phlebotomists at CHOP will draw blood. Subjects who are pregnant may not enroll or continue in the study. Therefore, a pregnancy test will be completed on all females 9 years of age or older at each visit that includes a DXA. Collection and storage of stool is associated with a small risk of fecal contamination. However, proper stool collection instructions and supplies (gloves, disposable collection containers, storage freezer container) will be provided for safety and convenience and, therefore, greatly reduce the risk of contamination. There is minimal risk associated with the radiation from DXA or pQCT. The known risks associated with MRI/MRS are minimal. Subjects may feel uncomfortable inside the magnet if they do not like to be inside small places or has difficulty lying still. Subjects may also hear loud noises while inside the magnet. The magnet is always on. Any metal objects on or inside the subject's body may heat up, move, and or/or not function properly within the scanning room. To minimize any risk from these objects, MRI/MRS personnel will screen subjects. Another risk is that from a metallic object flying through the air toward the magnet and hitting the subject. Safety measures in place to reduce this risk include screening all persons and materials entering the scanning room, and closing the study room door to minimize the risk of someone accidentally walking into or bringing an object into the magnet room. Only subjects 10 years of age or older will have MRIs and will not require sedation. In summary, the minimal risks associated with this study are blood, urine and stool samples, questions for dietary intake, demographics and disease severity, and minimal radiation exposure from DXA. Subjects and their families will be asked to participate for a total of 18 months.

2. LYM-X-SORB™ Supplement Safety Data 1987 to 2001: Animal Studies:
A single dose of the LYM-X-SORB™ eutectic was administered at dose volumes of 2.5, 5.0 or 10.0 ml/kg to groups of 10 male rats and a separate group was used as controls (McNeil Pharmaceutical, Spring House, PA 1987). The growth of rats (3, 7 and 14 days) administered LYM-X-SORB™ at 2.5 and 5 ml/kg was comparable to the control group. The largest dosed animals did show reduced growth. The only clinical sign in the 2.5 and 5 ml/kg treated rats was moist rales. Additional mild clinical observations (unkempt appearance in 8 of 10 rats, chromorhinorrhea in 3 of 10, urine stained abdominal hair in 5 of 10, decreased spontaneous motor activity in 5 of 10, ptosis in 5 of 10, and pale extremities in 1 of 10) were noted for the 10 ml/kg dose.

Individual beagle dogs (4 males and 4 females) were administered a single dose of encapsulated LYM-X-SORB™ at 1.0 ml/kg (9-11 kg) and control dogs (2 of both sexes) were dosed with a comparable number of

empty hard gelatin capsules (McNeil Pharmaceutical, Spring House, PA, 1987). Recorded observations were made at 3, 7, 10, and 14 days post dosing. No deaths occurred in either group and weights were maintained for all animals. All LYM-X-SORB™ animals were devoid of any clinical signs.

A morphology study of LYM-X-SORB™ in rats was conducted (Upjohn Company, Kalamazoo MI, 1990). Four groups of fasted rats received 1ml of (a) saline, (b) aqueous LYM-X-SORB™, (c) aqueous LYM-X-SORB™ or (d) 0.1M citric acid by injection into the duodenum through a midline incision. In order to observe immediate effects, portions of the jejunum were removed at 5-20 cm from the site of duodenal injection 15 minutes after administration. Intestinal cross sections were processed histologically. No significant differences were found in epithelial cell integrity after administration of LYM-X-SORB™ or LYM-X-SORB™ compared to saline; whereas, 0.1M citric acid resulted in loss of epithelial cells and other abnormal effects.

A study of multiple dosing to pregnant rats and their male offspring was conducted (Guy Lepage, PhD, and Claude C. Roy, MD of Hopital Sainte Justine, Pediatric Research Center, Universite of Montreal, Montreal (Quebec) Canada, 1995-1996). LYM-X-SORB™ was fed as the only source of lipids (22% by weight in a balanced diet) to fifteen pregnant rats a week prior to birth, and thereafter during lactation, also to selected male offspring (fifteen) until four months of age. Two control groups of rats were treated similarly except the source of fat was triglyceride (22% having a fatty acid profile similar to LYM-X-SORB™, or 5.5% in commercial chow). Weights of each animal, food intake over 3-4 days and any appearances of abnormal behaviors were noted weekly for each animal. LYM-X-SORB™ provided the caloric demands of pregnancy and lactation without any adverse effect. There were no statistical differences among the growth of the three groups of male rats and no adverse clinical observations were recorded. All animals were healthy, robust and active.

A 13-week oral toxicity study was conducted (ClinTrials BioResearch, Project No. 87680, Senneville (Quebec) Canada, 1997) to determine the potential chronic toxicity of LYM-X-SORB™ in comparison to triglyceride. A diet consisting of 20% fat (w/w) was administered to four groups of rats (N=20/sex/group). The percentage of LYM-X-SORB™ comprising the total fat concentration in the 4 groups was 0, 5, 10 and 20% and the corresponding levels of triglycerides was 20, 15, 10 and 0% respectively. The toxicological evaluations were unremarkable. There were no treatment-related clinical signs, no effects on body weights, and no deaths. Hematology, clinical chemistry and urinalysis were within normal parameters, as were gross and histological pathology and organ weight evaluations.

<u>Human Studies:</u>
In order to evaluate the extent of absorption of ^{13}C-labeled, LYM-X-SORB™ or triglyceride a crossover design was conducted in subjects with CF and healthy volunteers (SBIR Phase I: 1R43 DK 48208-01, Guy Lepage, PhD, Claude Roy, MD, of Hopital Sainte Justine, Pediatric Research Center, Universite of Montreal, Montreal (Quebec) Canada, and David W. Yesair, BioMolecular Products, Inc. Byfield MA, 1994). Decreased respiratory excretion rates of $^{13}CO_2$, without enzyme supplementation, indicate fat malabsorption. In the absence of pancreatic enzyme medication, the subjects with CF poorly absorbed ^{13}C-labeled triglycerides. However, in the ^{13}C-labeled LYM-X-SORB™, subjects with CF and healthy subjects had equivalent absorption, which was similar to ^{13}C-labeled triglycerides in healthy subjects. Thus, LYM-X-SORB™ represents a readily absorbable lipid matrix.

In a crossover design study and in the absence of pancreatic enzyme supplements, the acute oral absorption of LYM-X-SORB™, supplemented with added retinyl palmitate, was compared to that of Scandishake® with added retinyl palmitate (Guy Lepage, PhD, Claude Roy, MD, of Hopital Sainte Justine, Pediatric Research Center, Universite of Montreal, Montreal (Quebec) Canada) [7]. Approximately 29 g/m^2 of body surface area of LYM-X-SORB™ or triglyceride (Scandishake®) and 48,000 I.U./m^2 of body surface area of retinyl palmitate were orally consumed at time zero. In five subjects with CF and three controls (all fasted overnight), plasma triglyceride peaked at approximately 2 hours (Cmax) and decreased thereafter until 12 h (area under curve, AUC). Both Cmax and AUC for the LYM-X-SORB™ supplement were statistically greater by 10-fold than the corresponding values following the ingestion of Scandishake®. Similar 10 fold differences were observed for

the absorption of retinyl palmitate. Again, LYM-X-SORB™ was shown to be a readily absorbable lipid matrix and enhances the absorption of fat-soluble retinyl palmitate [7].

In a one year double blind feeding study (Guy Lepage, PhD, Claude Roy, MD, of Hopital Sainte Justine, Pediatric Research Center, Universite of Montreal, Montreal (Quebec) Canada, 1998-2001) [7], the daily consumption of LYM-X-SORB™ in comparison to triglyceride (24g of lipid per 1.72 m^2 of body surface) by subjects with CF (who also consumed, daily pancreatic enzyme supplement) had improved the clinical outcome. The LYM-X-SORB™ and triglyceride formulations contained at least 50% polyunsaturated fatty acid with a mole ratio of linolenic and α-linolenic fatty acids of about 5:1. LYM-X-SORB™ contained about 20% (w/w) LPC. Per protocol analysis of 48 subjects showed the LYM-X-SORB™ produced better clinical outcomes in comparison to triglyceride supplements as follows:

- Increased energy intake (~10%) from diet (P = 0.002)
- Increased plasma levels of linoleic acid (P <0.001) and α-linolenic acid (P <0.01)
- Increased plasma levels of vitamin E (P <0.001) and retinal-binding protein (P = 0.02)
- Increased growth in terms of weight (P <0.05) and height (P = 0.03) Z scores
- Improved lung function: FEV_1 (P = 0.02) and PE max (P = 0.02)

Liver and other clinical biochemistry did not change in the subjects. In conclusion, the readily absorbable LYM-X-SORB™ was an effective and safe medical food.

Risk for Adverse Events

In the 12-month Montreal study [7] of 73 subjects on LYM-X-SORB™ or a triglyceride placebo, adverse event data were collected and included in the final report (Protocol #960160) from Ferndale Labs, Inc. No adverse events were directly attributed to the LYM-X-SORB™ product. The most common adverse event was abdominal pain and was similar in both the LYM-X-SORB™ and triglyceride placebo groups. Other adverse events were rhinitis, diarrhea, eructation, cough, anorexia, nausea, headache, fever and vomiting, and were similar between the two groups. These symptoms are also common in people with CF. Of the reported adverse events, 81% were described as mild intensity, 18% moderate, 1% severe, and < 1% unknown. There were 42 serious adverse events reported by 22 subjects and similar between groups. All were attributed to a cause other than the study supplement, and generally, were related to the underlying CF. There were no deaths.

In summary, the risk of adverse events to our subjects taking LYM-X-SORB™ for 18 months is minimal.

Other: Confidentiality will be maintained by the use of subject code numbers, rather than names, in the database, presentations and publications. In the event of a serious adverse event during the study protocol, it will be documented and reported to the DSMB for discussion and review, and reported to the CHOP IRB and GCRC and the IRBs at other home CF Centers, and to all members of the research team, and to the NIH and Avanti Polar Lipids, Inc. The medical, physical and social risks are minimal for subjects participating in this study. The major principle behind this application is that many children with CF in the U.S. have abnormal EFA and choline status. The effects of supplementation of the organized lipid matrix LYM-X-SORB™ on improving EFA deficiency and choline status have yet to be established. Untreated EFA deficiency and continued malabsorption of fats in the diet likely contributes to the morbidity of CF. This study will provide information to determine if LYM-X-SORB™ may be an effective supplementation that will improve care for subjects with CF.

2. Adequacy of Protection Against Risks

a. Recruitment and Informed Consents

Subjects will be recruited from ten CF Centers after introduction by the subject's CF physician or nurse practitioner. A CHOP-based research team member will then continue the introduction of the study to the families of eligible subjects. All members of the team will be available to discuss the details and answer any study related questions as they arise (need to rework this for the other Centers).The Project Coordinator or Research Technician will obtain fully informed, written consent from the parent(s) or legal guardian(s) of the subjects and assent from the subjects after all questions have been answered. The families will be given written copies of all study materials. The investigators are well aware of the time and level of age-appropriate explanation required for child and adult subjects and their families to be fully informed.

b. Protection Against Risk
The risks of drawing blood are minimal, and include temporary discomfort from the needle stick, bruising and rarely an infection at the site. Each subject will have approximately no more than 5 Tbsp. of blood drawn at each study visit. Experienced GCRC pediatric research nurses and phlebotomists at CHOP will draw blood at the baseline, 3, 12 and 18 months visits to CHOP, and experienced nurses or phlebotomists will draw blood at the 6 months visit at the home CF Centers. There are minimal risks associated with DXA and pQCT scans.

The radiation exposure for each of the scans is summarized below, along with the dose for natural radiation exposure as a reference. The International Commission on Radiological Protection (ICRP) recommends calculating the effective dose equivalent (EDE) for each procedure. This measure incorporates weighting factors for the radiosensitivity of the different body tissues. The total EDE is the weighted sum of the effective dose to all organs irradiated by the scan.

**Source**	**Effective Dose Equivalent (μSv)**
**Natural Radiation Sources**	
Natural background radiation at sea level	3,000 per year
Roundtrip transcontinental airplane flight	60
**PQCT (Stratec 2000)**	
Tibia 3% site	< 0.01
Tibia 38 % site	< 0.01
Tibia 66% site	< 0.01
Radius 3% site	<0.01
Radius 30% site	<0.01
**DXA (Hologic QDR-4500)**	
Lumbar spine	3.8
Whole body	2.6

Subjects who are pregnant may not enroll or continue in the study. Therefore, a pregnancy test will be completed on all females 9 years of age or older at each visit that includes a DXA. Collection and storage of stool is associated with a small risk of fecal contamination. However, proper stool collection instructions and supplies (gloves, disposable collection containers, storage freezer container) will be provided for safety and convenience and, therefore, greatly reduce the risk of contamination. The known risks associated with MRI/MRS are minimal. Subjects may feel uncomfortable inside the magnet if they do not like to be inside small places or has difficulty lying still. Subjects may also hear loud noises while inside the magnet. The magnet is always on. Any metal objects on or inside the subject's body may heat up, move, and or/or not function properly within the scanning room. To minimize any risk from these objects, MRI/MRS personnel will screen subjects. Another risk is that from a metallic object flying through the air toward the magnet and hitting the subject. Safety measures in place to reduce this risk include screening all persons and materials entering the scanning room, and closing the study room door to minimize the risk of someone accidentally walking into or bringing an object into the magnet room. Only subjects 10 years of age or older will have MRIs and will not require sedation.

If a result from a research test unexpectedly appears to be important for immediate clinical care, these data will be provided to Drs. Stallings, Mascarenhas and Maqbool as the physician investigators. They will discuss with the subject/family and, with their permission, provide the information to the home CF Center clinical team. Access to the main study database is through password protection and enforcement of user privileges. Records of individuals are stored with ID numbers and a name code with no discernible personal identifiers. All research team members have completed the IRB investigator training.

All research personnel responsible for collecting data on the subjects will be blinded to the randomization throughout the study. Study supplement will be labeled with a code number. Two individuals at CHOP will be designated to have access to the randomization code. These two individuals will include a member of the Pharmacy staff and a physician trained in nutrition research and care who will not participate in any aspect of the study. In the event of a medical emergency, Dr. Stallings will be unblinded for that individual subject so that she can provide the information to the subject's care team at CHOP or at their home CF Center.

3. Potential Benefits of the Proposed Research to Subjects and Others
There is no direct benefit to the subjects participating in this study, however, there is potential significant indirect benefit to both subjects and others. This supplementation study in children and adolescents with CF may contribute to the improvement of clinical status in CF. Occasionally, some information from the study will be useful to the care of an individual subject (abnormal baseline blood value, abnormal soft tissue calcification on the DXA scan), but it will not be common. There is a possible benefit of a growth (height, weight, muscle mass) and pulmonary function in subjects who have suboptimal EFA or choline status during and after 18 months of supplementation. Another minor benefit of the study is the provision of food coupons to the subjects and families to help offset the participant burden.

There is a possible psychosocial benefit for the subject participants and their family, as they will contribute to a clinical research study important to the health of many people with CF all over the world. The major benefit is that information gained from each subject, combined with our other subjects, will determine if people with CF can be helped by supplementation with LYM-X-SORB™.

4. Importance of Knowledge to be Gained
If proven effective, LYM-X-SORB™ will quickly become available to treat EFA and choline deficiency, growth and developmental failure in people with CF. In the future, LYM-X-SORB™ may become the standard of care and prevent these and other fat malabsorption-related complications in patients with CF and PI. It is likely that adults with CF will also use the product if proven to be safe and effective in children and adolescents.

5. Women and Minority Inclusion
Female subjects ages 5.0 to 18.9 years of age (at enrollment) are well represented. CF is primarily a disease of people with European ancestry. There are no exclusions for ethnic/racial backgrounds.

6. Inclusion of children
The subjects will be children and adolescents in this study. Children and adolescents are a special class of subjects. This study does not involve any other special class of subjects.

7. Data and Safety Monitoring Plan
The CF Foundation is supporting this study by providing the Data Safety and Monitoring Board (DSMB) for this study. This ensures optimal oversight by people who are well versed in CF-specific clinical research. The CFF DSMB is composed of experts in bio-ethics, clinical trials, biostatistics, safety monitoring, CF clinical and basic science, and CF clinical care. For this study, a subcommittee will be formed from the DSMB, a Data Monitoring Committee (DMC) consisting of at least 3 DSMB members (at least 2 physicians experienced in treating CF and a biostatistician), as well as ad hoc members appropriate for the review of this specific protocol. The DMC reviews will be conducted on a regular basis as specified in the DMC charter developed between the study Investigators and the DSMB (i.e. every 6 months by conference call). The DMC will be responsible for reviewing clinical data relating to safety and efficacy, conduct and review interim analyses, and ensure the continued scientific validity and merit of the study. Serious adverse events will be monitored in real time by the DMC chair throughout the study. SAEs will be reported by the Principal Investigator or her designee to the DMC chair within 24 hours of learning of the event. SAEs will also be reported to the CHOP IRB and the home CF Center IRBs. All study withdrawals will be reported within 24 hours of learning of the event. Adverse event summaries will be reported monthly, along with summaries of enrollment, withdrawals and screen failures. Abnormal laboratory values and other safety data will be reported bi-annually. All IRB comments about the

study will be reported as well . The CHOP IRB and GCRC will also monitor safety, along with the study team throughout the study.

7. Data Sharing Plan

The data sharing will be managed by the PI of the clinical site, Virginia Stallings, MD, at e-mail address: stallingsv@email.chop.edu, Phone: (215) 590-1664; FAX: (215) 590-3804, and address: Abramson Research Center, Joseph Stokes Research Institute, Rm 139, 3615 Civic Center Boulevard, Philadelphia, PA 19104. The data will be shared four years after completion of the SBIR II project funding, as allowed by the SBIR Program.

Requests for data will be forwarded to Dr. Stallings. The request for data will be reviewed by Dr. Stallings, and Drs. Shaw and Yesair (or Dr. Shaw's or Yesair's designee) for the following commitments by the requesting PI:

1. commitment for use for not-for-profit research purposes that will likely contribute to the improved care of people with CF;
2. commitment to maintaining the data in a secure environment;
3. commitment to destroy or return the data after analysis or publication;
4. commitment to maintaining all appropriate regulatory requirements; and
5. commitment to acknowledge the source of the data (NIH grant support and research team) in all publications and presentations.

Dr. Stallings or her designee will supply the requesting PI the data and codebook in a user-friendly method (i.e. CD, secure web transmission) in a timely fashion. A data sharing agreement will be developed between CHOP/Dr. Stallings and the requesting institution / PI in compliance with all IRB, HIPAA, NIH and other applicable regulations at the time of the request.

A complete data set (all participating subjects) will be held by Avanti, as the PI of the SBIR, and by CHOP, as the SBIR clinical site. An agreement will be developed between Avanti and CHOP to allow CHOP ongoing use of the data for research and education not-for-profit purposes.

Table 7.

Targeted/Planned Enrollment Table

This report format should NOT be used for data collection from study participants.

Study Title: Organized Lipid Matrix: Fatty Acids and Choline in CF
Total Planned Enrollment: 78 subjects

TARGETED/PLANNED ENROLLMENT: Number of Subjects

Ethnic Category	Sex/Gender		
	Females	Males	Total
Hispanic or Latino	3	3	6
Not Hispanic or Latino	53	53	106
Ethnic Category Total of All Subjects*	56	56	112
**Racial Categories**			
American Indian/Alaska Native	0	0	0
Asian	0	0	0
Native Hawaiian or Other Pacific Islander	0	0	0
Black or African-American	3	3	6
White	53	53	103
Racial Categories: Total of All Subjects *	56	56	112

*The “Ethnic Category Total of All Subjects” must be equal to the “Racial Categories Total of All Subjects.”

Reference List

1. Cystic Fibrosis Foundation. Cystic Fibrosis Foundation, Patient Registry 2003. Bethesda, MD: Cystic Fibrosis Foundation, 2004

2. Cystic Fibrosis Foundation. Cystic Fibrosis Foundation, Patient Registry 2002. Bethesda, MD: Cystic Fibrosis Foundation, 2003

3. Freedman SD, Blanco PG, Zaman MM et al. Association of cystic fibrosis with abnormalities in fatty acid metabolism. N Engl J Med 2004;350(6):560-569.

4. Innis SM, Davidson AG, Chen A, Dyer R, Melnyk S, James SJ. Increased plasma homocysteine and S-adenosylhomocysteine and decreased methionine is associated with altered phosphatidylcholine and phosphatidylethanolamine in cystic fibrosis. J Pediatr 2003;143(3):351-356.

5. Chen A, Innis S. Assessment of phospholipid malabsorption by quantification of fecal phospholipid. J Pediatr Gastroenterol Nutr 2004;39(1):85-91.

6. Chen AH, Innis SM, Davidson AG, James SJ. Phosphatidylcholine and lysophosphatidylcholine excretion is increased in children with cystic fibrosis and is associated with plasma homocysteine, S-adenosylhomocysteine, and S-adenosylmethionine. Am J Clin Nutr 2005;81(3):686-691.

7. Lepage G, Yesair DW, Ronco N et al. Effect of an organized lipid matrix on lipid absorption and clinical outcomes in patients with cystic fibrosis. Journal of Pediatrics 2002;141(2):178-185.

8. Roulet M, Frascarolo P, Rappaz I, Pilet M. Essential fatty acid deficiency in well nourished young cystic fibrosis patients. Eur J Pediatr 1997;156(12):952-956.

9. Cystic Fibrosis Foundation, editor. Concepts in CF Care: Pediatric Nutrition for Patients with Cystic Fibrosis. 01 Mar 28; Bethesda, MD: 2001.

10. Kalivianakis M, Verkade HJ. The mechanisms of fat malabsorption in cystic fibrosis patients. Nutrition 1999;15(2):167-169.

11. KUO PT, HUANG NN, BASSETT DR. The fatty acid composition of the serum chylomicrons and adipose tissue of children with cystic fibrosis of the pancreas. J Pediatr 1962;60:394-403.

12. Farrell PM, Mischler EH, Engle MJ, Brown DJ, Lau SM. Fatty acid abnormalities in cystic fibrosis. Pediatr Res 1985;19(1):104-109.

13. Lloyd-Still JD, Johnson SB, Holman RT. Essential fatty acid status and fluidity of plasma phospholipids in cystic fibrosis infants. Am J Clin Nutr 1991;54(6):1029-1035.

14. van Egmond AW, Kosorok MR, Koscik R, Laxova A, Farrell PM. Effect of linoleic acid intake on growth of infants with cystic fibrosis. Am J Clin Nutr 1996;63(5):746-752.

15. Lepage G, Paradis K, Lacaille F et al. Ursodeoxycholic acid improves the hepatic metabolism of essential fatty acids and retinol in children with cystic fibrosis. J Pediatr 1997;130(1):52-58.

16. Lloyd-Still JD, Bibus DM, Powers CA, Johnson SB, Holman RT. Essential fatty acid deficiency and predisposition to lung disease in cystic fibrosis. Acta Paediatr 1996;85(12):1426-1432.

17. Strandvik B, Gronowitz E, Enlund F, Martinsson T, Wahlstrom J. Essential fatty acid deficiency in relation to genotype in patients with cystic fibrosis. J Pediatr 2001;139(5):650-655.

18. Gibson RA, Teubner JK, Haines K, Cooper DM, Davidson GP. Relationships between pulmonary function and plasma fatty acid levels in cystic fibrosis patients. J Pediatr Gastroenterol Nutr 1986;5(3):408-415.

19. Biggemann B, Laryea MD, Schuster A, Griese M, Reinhardt D, Bremer HJ. Status of plasma and erythrocyte fatty acids and vitamin A and E in young children with cystic fibrosis. Scand J Gastroenterol Suppl 1988;143:135-141.

20. Congden PJ, Bruce G, Rothburn MM et al. Vitamin status in treated patients with cystic fibrosis. Arch Dis Child 1981;56(9):708-714.

21. Farrell PM, Bieri JG, Fratantoni JF, Wood RE, di Sant'Agnese PA. The occurrence and effects of human vitamin E deficiency. A study in patients with cystic fibrosis. J Clin Invest 1977;60(1):233-241.

22. Freedman SD, Katz MH, Parker EM, Laposata M, Urman MY, Alvarez JG. A membrane lipid imbalance plays a role in the phenotypic expression of cystic fibrosis in cfter -/- mice. Proc Natl Acad Sci 1999;96(24):13995-14000.

23. Steinkamp G, Demmelmair H, Ruhl-Bagheri I, von der HH, Koletzko B. Energy supplements rich in linoleic acid improve body weight and essential fatty acid status of cystic fibrosis patients. J Pediatr Gastroenterol Nutr 2000;31(4):418-423.

24. Henderson WR, Jr., Astley SJ, McCready MM et al. Oral absorption of omega-3 fatty acids in patients with cystic fibrosis who have pancreatic insufficiency and in healthy control subjects. J Pediatr 1994;124(3):400-408.

25. McKeon-O'Malley C, Siwek D, Lamoureux JA, Williams CL, Kowall NW. Prenatal choline deficiency decreases the cross-sectional area of cholinergic neurons in the medial septal nucleus. Brain Res 2003;977(2):278-283.

26. Brandner C. Perinatal choline treatment modifies the effects of a visuo-spatial attractive cue upon spatial memory in naive adult rats. Brain Res 2002;928(1-2):85-95.

27. Institute of Medicine. Dietary reference intakes for thiamin, riboflavin, niacin, vitamin B6, folate, vitamin B12, pantothenic acid, biotin, and choline. Washington, DC: National Academy Press; 1998.

28. Yen CL, Mar MH, Craciunescu CN, Edwards LJ, Zeisel SH. Deficiency in methionine, tryptophan, isoleucine, or choline induces apoptosis in cultured cells. J Nutr 2002;132(7):1840-1847.

29. James SJ, Miller BJ, Basnakian AG, Pogribny IP, Pogribna M, Muskhelishvili L. Apoptosis and proliferation under conditions of deoxynucleotide pool imbalance in liver of folate/methyl deficient rats. Carcinogenesis 1997;18(2):287-293.

30. da Costa KA, Cochary EF, Blusztajn JK, Garner SC, Zeisel SH. Accumulation of 1,2-sn-diradylglycerol with increased membrane-associated protein kinase C may be the mechanism for spontaneous hepatocarcinogenesis in choline-deficient rats. J Biol Chem 1993;268(3):2100-2105.

31. Handler P, Bernheim F. Choline deficiency in the hamster. Proc Soc Exptl Med 1949;72:569.

32. Buchman AL, Dubin MD, Moukarzel AA et al. Choline deficiency: a cause of hepatic steatosis during parenteral nutrition that can be reversed with intravenous choline supplementation. Hepatology 1995;22(5):1399-1403.

33. Mason JB. Biomarkers of nutrient exposure and status in one-carbon (methyl) metabolism. J Nutr 2003;133 Suppl 3:941S-947S.

34. Roy CC, Weber AM, Morin CL et al. Hepatobiliary disease in cystic fibrosis: a survey of current issues and concepts. J Pediatr Gastroenterol Nutr 1982;1(4):469-478.

35. Innis SM, Davidson A, Chen A, Dyer RA. Malabsorption of phosphatidylcholine and lysophosphatidylcholine occur concurrently with altered phospholipid and thiol metabolism in children with cystic fibrosis. Pediatr.Pulmonol. Suppl 25. 2003.

Ref Type: Abstract

36. Weizman Z, Durie PR, Kopelman HR, Vesely SM, Forstner GG. Bile acid secretion in cystic fibrosis: evidence for a defect unrelated to fat malabsorption. Gut 1986;27(9):1043-1048.

37. Roy CC, Weber AM, Morin CL et al. Abnormal biliary lipid composition in cystic fibrosis. Effect of pancreatic enzymes. N Engl J Med 1977;297(24):1301-1305.

38. Weber AM, Roy CC, Morin CL, Lasalle R. Malabsorption of bile acids in children with cystic fibrosis. N Engl J Med 1973;289(19):1001-1005.

39. Gaskin K, Nouri-Sorkhabi MH, Gruca MA, Kuchel PW. Intestinal phospholipid metabolism: its influence on fat digestion and absorption. Pediatric Pulmonology Supplement 22, S11.2. 2001.

Ref Type: Abstract

40. Ulane MM, Butler JD, Peri A, Miele L, Ulane RE, Hubbard VS. Cystic fibrosis and phosphatidylcholine biosynthesis. Clin Chim Acta 1994;230(2):109-116.

41. Lindblad A, Glaumann H, Strandvik B. Natural history of liver disease in cystic fibrosis. Hepatology 1999;30(5):1151-1158.

42. Colombo C, Battezzati PM. Hepatobiliary manifestations of cystic fibrosis. Eur J Gastroenterol Hepatol 1996;8(8):748-754.

43. Sokol RJ, Durie PR. Recommendations for management of liver and biliary tract disease in cystic fibrosis. Cystic Fibrosis Foundation Hepatobiliary Disease Consensus Group. J Pediatr Gastroenterol Nutr 1999;28 Suppl 1:S1-13.

44. Fishbein MH, Stevens WR. Rapid MRI using a modified Dixon technique: a non-invasive and effective method for detection and monitoring of fatty metamorphosis of the liver. Pediatr Radiol 2001;31(11):806-809.

45. Abdelmalek MF, Angulo P, Jorgensen RA, Sylvestre PB, Lindor KD. Betaine, a promising new agent for patients with nonalcoholic steatohepatitis: results of a pilot study. Am J Gastroenterol 2001;96(9):2711-2717.

46. Szczepaniak LS, Nurenberg P, Leonard D et al. Magnetic resonance spectroscopy to measure hepatic triglyceride content: prevalence of hepatic steatosis in the general population. Am J Physiol Endocrinol Metab 2005;288(2):E462-E468.

47. Thomas EL, Hamilton G, Patel N et al. Hepatic triglyceride content and its relation to body adiposity: a magnetic resonance imaging and proton magnetic resonance spectroscopy study. Gut 2005;54(1):122-127.

48. Machann J, Thamer C, Schnoedt B et al. Hepatic lipid accumulation in healthy subjects: a comparative study using spectral fat-selective MRI and volume-localized 1H-MR spectroscopy. Magn Reson Med 2006;55(4):913-917.

49. Boesch C, Machann J, Vermathen P, Schick F. Role of proton MR for the study of muscle lipid metabolism. NMR Biomed 2006;19(7):968-988.

50. Moghaddami N, Costabile M, Grover PK et al. Unique effect of arachidonic acid on human neutrophil TNF receptor expression: up-regulation involving protein kinase C, extracellular signal-regulated kinase, and phospholipase A2. J Immunol 2003;171(5):2616-2624.

51. Miele L, Cordella-Miele E, Xing M, Frizzell R, Mukherjee AB. Cystic fibrosis gene mutation (deltaF508) is associated with an intrinsic abnormality in Ca2+-induced arachidonic acid release by epithelial cells. DNA Cell Biol 1997;16(6):749-759.

52. Levy E, Gurbindo C, Lacaille F, Paradis K, Thibault L, Seidman E. Circulating tumor necrosis factor-alpha levels and lipid abnormalities in patients with cystic fibrosis. Pediatr Res 1993;34(2):162-166.

53. Rallidus LS, Aschos G, Liakos LK, Velissaridou AH, Anastasiadis G, Zampelas A. Dietary alpha linoleic decreases C-reactive protein, serum amyloid A and interleukin 6 in dyslipidaemic patients. Atherosclerosis 2003;167:237-242.

54. De Vizia B, Raia V, Spano C, Pavlidis C, Coruzzo A, Allesio M. Effect of an 8-month treatment with omega-3 fatty acids (eicosapentaenoic and docosahexaenoic) in patients with cystic fibrosis. J Parenter Enteral Nutr 2003;27(1):52-57.

55. Kurlandsky LE, Bennink MR, Webb PM, Ulrich PJ, Baer LJ. The absorption and effect of dietary supplementation with omega-3 fatty acids on serum leukotriene B4 in patients with cystic fibrosis. Pediatr Pulmonol 1994;18(4):211-217.

56. Tso P, Lam J, Simmonds WJ. The importance of the lysophosphatidylcholine and choline moiety of bile phosphatidylcholine in lymphatic transport of fat. Biochim Biophys Acta 1978;528(3):364-372.

57. Nilsson A, Borgstrom B. Absorption and metabolism of lecithin and lysolecithin by intestinal slices. Biochim Biophys Acta 1967;137(2):240-254.

58. Nouri-Sorkhabi MH, Chapman BE, Kuchel PW, Gruca MA, Gaskin KJ. Parallel secretion of pancreatic phospholipase A(2), phospholipase A(1), lipase, and colipase in children with exocrine pancreatic dysfunction. Pediatr Res 2000;48(6):735-740.

59. Yesair DW. Bicarbonate colloidal particles for delivering drugs or calories. United States. patent 5,891,466. 1999 Apr 6.

60. Yesair DW, Lepage G, Roy CC. Carbon-13 lipids, oral absorption in cystic fibrosis patients. 1994.
Ref Type: Generic

61. Lloyd-Still JD. Essential fatty acid deficiency and nutritional supplementation in cystic fibrosis. J Pediatr 2002;141(2):157-159.

62. Super M. Milestones in cystic fibrosis. Br Med Bull 1992;48(4):717-737.

63. Sheppard DN, Welsh MJ. Structure and function of the CFTR chloride channel. Physiol Rev 1999;79(1 Suppl):S23-S45.

64. O'Riordan CR, Erickson A, Bear C et al. Purification and characterization of recombinant cystic fibrosis transmembrane conductance regulator from Chinese hamster ovary and insect cells. J Biol Chem 1995;270(28):17033-17043.

65. Cobb BR, Ruiz F, King CM et al. A(2) adenosine receptors regulate CFTR through PKA and PLA(2). Am J Physiol Lung Cell Mol Physiol 2002;282(1):L12-L25.

66. Cobb BR, Hong JS, Clancy JP. PLA2 signaling governs A2B adenosine receptor activation of CFTR. Pediatric Pulmonology Suppl 20, 191. 2000.
Ref Type: Abstract

67. Eidelman O, BarNoy S, Razin M et al. Role for phospholipid interactions in the trafficking defect of Delta F508-CFTR. Biochemistry 2002;41(37):11161-11170.

68. Weber AM, Roy CC, Chartrand L et al. Relationship between bile acid malabsorption and pancreatic insufficiency in cystic fibrosis. Gut 1976;17(4):295-299.

69. Goodchild MC, Murphy GM, Howell AM, Nutter SA, Anderson CM. Aspects of bile acid metabolism in cystic fibrosis. Arch Dis Child 1975;50(10):769-778.

70. Roller RJ, Kern F. Minimal bile acid malabsorption and normal bile acid breath tests in cystic fibrosis and acquired pancreatic insufficiency. Gastroenterology 1977;72(4 Pt 1):661-665.

71. Harries JT, Muller DP, McCollum JP, Lipson A, Roma E, Norman AP. Intestinal bile salts in cystic fibrosis: studies in the patient and experimental animal. Arch Dis Child 1979;54(1):19-24.

72. Berge Henegouwen GP, van der Werf SD, Ruben AT. Fatty acid composition of phospholipids in bile in man: promoting effect of deoxycholate on arachidonate. Clin Chim Acta 1987;165(1):27-37.

73. Entenman C, Holloway RJ, Albright ML, Leong GF. Bile acids and lipid metabolism. II. Essential role of bile acids in bile phospholipid excretion. Arch Biochem Biophys 1969;130(1):253-256.

74. Nilsson S, Schersten T. Importance of bile acids for phospholipid secretion into human hepatic bile. Gastroenterology 1969;57(5):525-532.

75. Nilsson S, Schersten T. Influence of bile acids on the synthesis of biliary phospholipids in man. Eur J Clin Invest 1970;1(2):109-111.

76. Schersten T, Nilsson S, Cahlin E, Filipson M, Brodin-Persson G. Relationship between the biliary excretion of bile acids and the excretion of water, lecithin, and cholesterol in man. Eur J Clin Invest 1971;1(4):242-247.

77. Schersten T. Bile acids as a determining factor for synthesis and excretion of human bile lecithin. Helv Med Acta 1973;37(2):161-168.

78. LeBlanc MJ, Gavino V, Perea A, Yousef IM, Levy E, Tuchweber B. The role of dietary choline in the beneficial effects of lecithin on the secretion of biliary lipids in rats. Biochim Biophys Acta 1998;1393(2-3):223-234.

79. Smit JJ, Schinkel AH, Oude Elferink RP et al. Homozygous disruption of the murine mdr2 P-glycoprotein gene leads to a complete absence of phospholipid from bile and to liver disease. Cell 1993;75(3):451-462.

80. Wibom R, Hultman E. ATP production rate in mitochondria isolated from microsamples of human muscle. Am J Physiol 1990;259(2 Pt 1):E204-E209.

81. Blomstrand E, Ekblom B, Newsholme EA. Maximum activities of key glycolytic and oxidative anzymes in human muscle from differently trained individuals. J Physiol Lond 1986;381:111-118.

82. Holloszy JO. Biochemical adaptations in muscle. Effects of exercise on mitochondrial oxygen uptake and respiratory enzyme activity in skeletal muscle. J Biol Chem 1967;242(9):2278-2282.

83. von Ruecker AA, Bertele R, Harms HK. Calcium metabolism and cystic fibrosis: mitochondrial abnormalities suggest a modification of the mitochondrial membrane. Pediatr Res 1984;18(7):594-599.

84. Orenstein DM, Franklin BA, Doershuk CF et al. Exercise conditioning and cardiopulmonary fitness in cystic fibrosis. The effects of a three-month supervised running program. Chest 1981;80(4):392-398.

85. Salh W, Bilton D, Dodd M, Webb AK. Effect of exercise and physiotherapy in aiding sputum expectoration in adults with cystic fibrosis. Thorax 1989;44(12):1006-1008.

86. Nixon PA, Orenstein DM, Kelsey SF, Doershuk CF. The prognostic value of exercise testing in patients with cystic fibrosis. N Engl J Med 1992;327(25):1785-1788.

87. Blair SN, Kohl HW, III, Paffenbarger RS, Jr., Clark DG, Cooper KH, Gibbons LW. Physical fitness and all-cause mortality. A prospective study of healthy men and women. JAMA 1989;262(17):2395-2401.

88. Ward SA, Tomezsko JL, Holsclaw DS, Paolone AM. Energy expenditure and substrate utilization in adults with cystic fibrosis and diabetes mellitus. Am J Clin Nutr 1999;69(5):913-919.

89. Buchdahl RM, Cox M, Fulleylove C et al. Increased resting energy expenditure in cystic fibrosis. J Appl Physiol 1988;64(5):1810-1816.

90. Thomson MA, Wilmott RW, Wainwright C, Masters B, Francis PJ, Shepherd RW. Resting energy expenditure, pulmonary inflammation, and genotype in the early course of cystic fibrosis. J Pediatr 1996;129(3):367-373.

91. Fried MD, Durie PR, Tsui LC, Corey M, Levison H, Pencharz PB. The cystic fibrosis gene and resting energy expenditure. J Pediatr 1991;119(6):913-916.

92. Pencharz PB, Durie PR. Pathogenesis of malnutrition in cystic fibrosis, and its treatment. Clin Nutr 2000;19(6):387-394.

93. Bent ED, Bell JD. Quantification of the interactions among fatty acid, lysophosphatidylcholine, calcium, dimyrstophosphatidylcholine vesicles and phospholipase A2. Biochim Biophys 1995;1254(3):349-360.

94. Yesair DW, Shaw WA, Burgess SW, McKee RT. Modification of solid 3-Sn-phosphoglycerides. Patent Cooperation Treaty (PCT). United States. patent International Publication Number WO 03/031593 A2. 2003.

95. Lands L, Desmond KJ, Demizio D, Pavilanis A, Coates AL. The effects of nutritional status and hyperinflation on respiratory muscle strength in children and young adults. Am Rev Respir Dis 1990;141(6):1506-1509.

96. Hart N, Tounian P, Clement A et al. Nutritional status is an important predictor of diaphragm strength in young patients with cystic fibrosis. Am J Clin Nutr 2004;80(5):1201-1206.

97. Lands LC, Heigenhauser GJ, Jones NL. Respiratory and peripheral muscle function in cystic fibrosis. Am Rev Respir Dis 1993;147(4):865-869.

98. ATS/ERS Statement on respiratory muscle testing. Am J Respir Crit Care Med 2002;166(4):518-624.

99. Seid M, Varni JW, Segall D, Kurtin PS. Health-related quality of life as a predictor of pediatric healthcare costs: a two-year prospective cohort analysis. Health Qual Life Outcomes 2004;2:48.

100. Quittner AL. Measurement of quality of life in cystic fibrosis. Curr Opin Pulm Med 1998;4(6):326-331.

101. Quittner AL, Buu A, Messer MA, Modi AC, Watrous M. Development and validation of The Cystic Fibrosis Questionnaire in the United States: a health-related quality-of-life measure for cystic fibrosis. Chest 2005;128(4):2347-2354.

102. Aris RM, Merkel PA, Bachrach LK et al. Guide to bone health and disease in cystic fibrosis. J Clin Endocrinol Metab 2005;90(3):1888-1896.

103. Rovner AJ, Zemel BS, Leonard MB, Schall JI, Stallings VA. Mild to moderate cystic fibrosis is not associated with increased fracture risk in children and adolescents. J Pediatr 2005;147:327-331.

104. Baggio B. Fatty acids, calcium and bone metabolism. J Nephrol 2002;15(6):601-604.

105. Watkins BA, Li Y, Seifert MF. Nutraceutical fatty acids as biochemical and molecular modulators of skeletal biology. J Am Coll Nutr 2001;20(5 Suppl):410S-416S.

106. Albertazzi P, Coupland K. Polyunsaturated fatty acids. Is there a role in postmenopausal osteoporosis prevention? Maturitas 2002;42(1):13-22.

107. Kruger MC, Coetzer H, de Winter R, Gericke G, van Papendorp DH. Calcium, gamma-linolenic acid and eicosapentaenoic acid supplementation in senile osteoporosis. Aging (Milano) 1998;10(5):385-394.

108. Vanpapendorp DH, Coetzer H, Kruger MC. Biochemical profile of osteoporotic patients on essential fatty-acids supplement. Nutr Res 1995;15:325-334.

109. Bassey EJ, Littlewood JJ, Rothwell MC, Pye DW. Lack of effect of supplementation with essential fatty acids on bone mineral density in healthy pre- and postmenopausal women: two randomized controlled trials of Efacal v. calcium alone. Br J Nutr 2000;83(6):629-635.

110. Dunbar-Jacob J, Dunning EJ, Dwyer K. Compliance research in pediatric and adolescent populations: two decades of research. In: Krasnegor NP, Epstein L, Johnson SB, Yaffe SJ, editors. Development Aspects of Health Compliance Behavior. Hillside, NJ: Erlbaum; 1993:29-51.

111. Anthony H, Paxton S, Bines J, Phelan P. Psychosocial predictors of adherence to nutritional recommendations and growth outcomes in children with cystic fibrosis. J Psychosom Res 1999;47(6):623-634.

112. Lemanek KL, Kamps J, Chung NB. Empirically supported treatments in pediatric psychology: regimen adherence. J Pediatr Pscyh 2001;26(5):253-275.

113. Weinstein AG, Cuskey W. Theophylline compliance in asthmatic children. Ann Allergy 1985;54:19-24.

114. da Costa IG, Rapoff MA, Lemanek K, Goldstein GL. Improving adherence to medication regimens for children with asthma and its effects on clinical outcome. J Appl Beh Analysis 1997;30:687-691.

115. Wysocki T, Green LB, Huxtable K. Blood glucose monitoring by diabetic adolescents: compliance and metabolic control. Health Psychol 1989;8:267-284.

116. Tucker CM, Petersen S, Herman KC et al. Self-regulation predictors or medication adherence among ethnically different pediatric patients with renal transplants. J Pediatr Psych 2001;26(8):455-464.

117. Rapoff MA. Adherence of pediatric medical regimens. New York: Kluwer/Plenum; 1999.

118. Lemanek KL. Adherence issues in medical management of asthma. J Pediatr Psych 1990;15:437-458.

119. Rapoff MA, Christophersen ER. Compliance of pediatric patients with medical regimens: a review and evaluation. In: Stuart RB, editor. Adherence, Compliance and Generalization in Behavioral Medicine. New York: Brunner/Mazel; 1982:79-124.

120. Stark LJ, Bowen AM, Tyc VL, Evans S, Passero MA. A behavioral approach to increasing calorie consumption in children with cystic fibrosis. J Pediatr Psychol 1990;15(3):309-326.

121. Stark LJ, Knapp L, Bowen AM et al. Behavioral treatment of calorie consumption in children with cystic fibrosis: replication with two year follow-up. J Appl Behav Anal 1993;26:435-450.

122. Stark LJ, Mulvihill MM, Powers SW et al. Behavioral intervention to improve calorie intake of children with cystic fibrosis: Treatment versus wait list control. J Pediatr Gastroenterol Nutr 1996;22(3):240-253.

123. Stark LJ, Hommel KA, Mackner LM et al. Randomized trial comparing two methods of increasing dietary calcium intake in children with inflammatory bowel disease. J Pediatr Gastroenterol Nutr 2005;in press.

124. Stark LJ, Janicke DM, McGrath AM, Hommel KA, Lovell D. Prevention of osteoporosis: a randomized clinical trial to increase calcium intake in children with juvenile rheumatoid arthritis. J Pediatr Psychol 2005;in press.

125. Wu GD, Chen J, Hoffmann C et al. Linking long-term dietary patterns with gut microbial enterotypes. Science 2011;334(6052):105-108.

126. Spencer MD, Hamp TJ, Reid RW, Fischer LM, Zeisel SH, Fodor AA. Association between composition of the human gastrointestinal microbiome and development of fatty liver with choline deficiency. Gastroenterology 2011;140(3):976-986.

127. Yesair DW. Composition for delivery of orally administered drugs and other substances. United States. patent 4,874,795. 2004.

128. Lepage G, Yesair DW, Champagne J, Ronco N, Bureau N, Roy CC. Normalization of the absorption of lipids and of Vitamin A from the matrix LYM-X-SORB with added retinly palmitate in CF patients. Pediatric Pulmonology Supplement 19[September], 303. 1999.
Ref Type: Abstract

129. Yesair DW. Method for making lysophosphatidylcholine. United States. patent 5,716,814. 2004.

130. Yesair DW, Shaw WA, Burgess SW, McKee RT. Modification of solid 3-Sn-phosphoglycerides. Patent Cooperation Treaty (PCT) WO 03/031593. United States. patent International Publication Number WO 03/031593 A2. 2003 Apr 17.

131. Borowitz D, Baker RD, Stallings V. Consensus report on nutrition for pediatric patients with cystic fibrosis. J Pediatr Gastroenterol Nutr 2002;35(3):246-259.

132. Tomezsko JL, Stallings VA, Scanlin TF. Dietary intake of healthy children with cystic fibrosis compared with normal control children. Pediatrics 1992;90(4):547-553.

133. Zemel BS, Kawchak DA, Cnaan A, Zhao H, Scanlin TF, Stallings VA. Prospective evaluation of resting energy expenditure, nutritional status, pulmonary function, and genotype in children with cystic fibrosis. Pediatr Res 1996;40(4):578-586.

134. Tomezsko JL, Stallings VA, Kawchak DA, Goin JE, Diamond G, Scanlin TF. Energy expenditure and genotype of children with cystic fibrosis. Pediatr Res 1994;35(4 Pt 1):451-460.

135. Tomezsko JL, Scanlin TF, Stallings VA. Body composition of children with cystic fibrosis with mild clinical manifestations compared with normal children. Am J Clin Nutr 1994;59(1):123-128.

136. Stettler N, Kawchak DA, Boyle LL et al. Prospective evaluation of growth, nutritional status, and body composition in children with cystic fibrosis. Am J Clin Nutr 2000;72(2):407-413.

137. Stallings VA, Fung EB, Hofley PM, Scanlin TF. Acute pulmonary exacerbation is not associated with increased energy expenditure in children with cystic fibrosis. J Pediatr 1998;132(3 Pt 1):493-499.

138. Stallings VA, Tomezsko JL, Schall JI et al. Adolescent development and energy expenditure in females with cystic fibrosis. Clin Nutr 2005;24:737-745.

139. Fung EB, Barden EM, Wasserman D et al. A six-month study of growth and energy expenditure in children with cystic fibrosis taking a pulmonary inhalation medication (rhDNase). J Am Coll Nutr 1999;18(4):330-338.

140. Zemel BS, Jawad AF, FitzSimmons S, Stallings VA. Longitudinal relationship among growth, nutritional status, and pulmonary function in children with cystic fibrosis: analysis of the Cystic Fibrosis Foundation National CF Patient Registry. J Pediatr 2000;137(3):374-380.

141. Cohen JR, Schall JI, Ittenbach RF, Zemel BS, Scanlin TF, Stallings VA. Pancreatic status verification in children with cystic fibrosis: fecal elastase status predicts prospective changes in nutritional status. J Pediatr Gastroenterol Nutr 2005;40:438-444.

142. Birch LL. Effects of peer models' food choices and eating behaviors on preschoolers' food preferences. Child Development 1980;51:489-496.

143. Birch LL. Dimensions of preschool children's food preferences. Journal of Nutrition Education 1979;11:77-80.

144. Birch LL. Preschool children's food preferences and consumption patterns. Journal of Nutrition Education 1979;11:189-192.

145. Birch LL, Birch D, Marlin DW, Kramer L. Effects of instrumental consumption on children's food preference. Appetite 1982;3(2):125-134.

146. Kuczmarski RJ, Ogden CL, Grummer-Strawn LM et al. CDC growth charts: United States. Adv Data 2000;(314):1-27.

147. Koc H, Mar MH, Ranasinghe A, Swenberg JA, Zeisel SH. Quantitation of choline and its metabolites in tissues and foods by liquid chromatography/electrospray ionization-isotope dilution mass spectrometry. Anal Chem 2002;74(18):4734-4740.

148. Han X, Gross RW. Electrospray ionization mass spectroscopic analysis of human erythrocyte plasma membrane phospholipids. Proc Natl Acad Sci U S A 1994;91(22):10635-10639.

149. Han X. Characterization and direct quantitation of ceramide molecular species from lipid extracts of biological samples by electrospray ionization tandem mass spectrometry. Anal Biochem 2002;302(2):199-212.

150. Blom TS, Koivusalo M, Kuismanen E, Kostiainen R, Somerharju P, Ikonen E. Mass spectrometric analysis reveals an increase in plasma membrane polyunsaturated phospholipid species upon cellular cholesterol loading. Biochemistry 2001;40(48):14635-14644.

151. Liebisch G, Drobnik W, Lieser B, Schmitz G. High-throughput quantification of lysophosphatidylcholine by electrospray ionization tandem mass spectrometry. Clin Chem 2002;48(12):2217-2224.

152. BLIGH EG, DYER WJ. A rapid method of total lipid extraction and purification. Can J Med Sci 1959;37(8):911-917.

153. Agren JJ, Julkunen A, Penttila I. Rapid separation of serum lipids for fatty acid analysis by a single aminopropyl column. J Lipid Res 1992;33(12):1871-1876.

154. Duffin K, Obukowicz M, Raz A, Shieh JJ. Electrospray/tandem mass spectrometry for quantitative analysis of lipid remodeling in essential fatty acid deficient mice. Anal Biochem 2000;279(2):179-188.

155. Brugger B, Erben G, Sandhoff R, Wieland FT, Lehmann WD. Quantitative analysis of biological membrane lipids at the low picomole level by nano-electrospray ionization tandem mass spectrometry. Proc Natl Acad Sci U S A 1997;94(6):2339-2344.

156. Koivusalo M, Haimi P, Heikinheimo L, Kostiainen R, Somerharju P. Quantitative determination of phospholipid compositions by ESI-MS: effects of acyl chain length, unsaturation, and lipid concentration on instrument response. J Lipid Res 2001;42(4):663-672.

157. DeLong CJ, Baker PR, Samuel M, Cui Z, Thomas MJ. Molecular species composition of rat liver phospholipids by ESI-MS/MS: the effect of chromatography. J Lipid Res 2001;42(12):1959-1968.

158. Hollis BW, Kamerud JQ, Selvaag SR, Lorenz JD, Napoli JL. Determination of vitamin D status by radioimmunoassay with an 125I-labeled tracer. Clin Chem 1993;39(3):529-533.

159. Hollis BW, Kamerud JQ, Kurkowski A, Beaulieu J, Napoli JL. Quantification of circulating 1,25-dihydroxyvitamin D by radioimmunoassay with 125I-labeled tracer. Clin Chem 1996;42(4):586-592.

160. McDonald JG, Thompson BM, McCrum EC, Russell DW. Extraction and analysis of sterols in biological matrices by high performance liquid chromatography electrospray ionization mass spectrometry. Methods Enzymol 2007;432:145-170.

161. Institute of Medicine. Dietary reference intakes for vitamin A, vitamin K, boron, chromium, copper, iodine, iron, manganese, molybdenum, nickel, vanadium, and zinc. Washington DC: National Academy Press; 2001.

162. Hauschka PV, Lian JB, Cole DE, Gundberg CM. Osteocalcin and matrix Gla protein: vitamin K-dependent proteins in bone. Physiol Rev 1989;69(3):990-1047.

163. Gallop PM, Lian JB, Hauschka PV. Carboxylated calcium-binding proteins and vitamin K. N Engl J Med 1980;302(26):1460-1466.

164. Farrugia W, Melick RA. Metabolism of osteocalcin. Calcif Tissue Int 1986;39(4):234-238.

165. Bikle DD. Biochemical markers in the assessment of bone disease. Am J Med 1997;103(5):427-436.

166. Juppner H, Brown E, Kronenberg H. Parathyroid hormone. In: Favus, editor. Primer on the Metabolic Bone Diseases and Disorders of Mineral Metabolism. Philadelphia, PA: Lippincott, Williams, & Wilkins; 1999:80-87.

167. Price CP. Multiple forms of human serum alkaline phosphatase: detection and quantitation. Ann Clin Biochem 1993;30 (Pt 4):355-372.

168. Kosla SKM. Biochemical markers of bone turnover. In: Favus, editor. Primer on the Metabolic one Diseases and Disorders of Mineral Metabolism. Philadelphia, PA: Lippincott, Williams & Wilkins; 1999:128-134.

169. Morris A, Kanner RE, Crapo R, Gardner RM. Clinical Pulmonary Function Testing: A Manual of Uniform Laboratory Procedures. 2nd ed. Salt Lake City, UT: Intermountain Thoracic Society; 1984.

170. Gardner RM. Report of Snowbird workshop on standardization of spirometry. Am Rev Resp Dis 1979;119:831-838.

171. Zapletal A. Lung Function in Children and Adolescents - Methods, Reference Values. In: Herzog H, editor. Progress in Respiration Research. Pratteln, Switzerland: Offsetdruck; 1987.

172. Hayot M, Guillaumont S, Ramonatxo M, Voisin M, Prefaut C. Determinants of the tension-time index of inspiratory muscles in children with cystic fibrosis. Pediatr Pulmonol 1997;23(5):336-343.

173. Weir JB. New methods for calculating metabolic rate with special reference to protein metabolism. 1949. Nutrition 1990;6(3):213-221.

174. FAO/WHO/UNU Expert Consultation. Energy and protein requirements: technical report series. 1985724.)

175. Schofield WN. Predicting basal metabolic rate, new standards and review of previous work. Hum Nutr Clin Nutr 1985;39 Suppl 1:5-41.

176. Freskanich D, Buzzard IM, Welch BT et al. Comparison of a computerized and a manual method of food coding for nutrient intake studies. J Am Diet Assoc 1988;88:1263-1267.

177. Institute of Medicine. Energy, carbohydrate, fiber, fat, fatty acids, cholesterol, protein and amino acids. Washington, D.C.: National Academies Press; 2002.

178. Lohman TG, Roche AR, Martorell R. Anthropometric Standardization Reference Manual. Champaign, IL: Human Kinetics; 1988.

179. Himes JH, Roche AF, Thissen D, Moore WM. Parent-specific adjustments for evaluation of recumbent length and stature of children. Pediatr 1985;75:304-313.

180. Brook CGD. Determination of Body Composition of Children from Skinfold Measurements. Archives of Disease in Childhood 1971;46(246):182-&.

181. Slaughter MH, Lohman TG, Boileau RA et al. Skinfold Equations for Estimation of Body Fatness in Children and Youth. Human Biology 1988;60(5):709-723.

182. Wahner HW, Looker A, Dunn WL, Walters LC, Hauser MF, Novak C. Quality control of bone densitometry in a national health survey (NHANES III) using three mobile examination centers. J Bone Miner Res 1994;9(6):951-960.

183. Faulkner KG, Gluer CC, Grampp S, Genant HK. Cross-calibration of liquid and solid QCT calibration standards: corrections to the UCSF normative data. Osteoporos Int 1993;3(1):36-42.

184. Louis O, Soykens S, Willnecker J, Van den WP, Osteaux M. Cortical and total bone mineral content of the radius: accuracy of peripheral computed tomography. Bone 1996;18(5):467-472.

185. Braun MJ, Meta MD, Schneider P, Reiners C. Clinical evaluation of a high-resolution new peripheral quantitative computerized tomography (pQCT) scanner for the bone densitometry at the lower limbs. Phys Med Biol 1998;43(8):2279-2294.

186. Schiessl H, Ferretti JL, Tsarczyk-Niemeyer G, Willnecker J. Noninvasive bone srength index as analyzed by peripheral quantitative computed tomography. In: Schonau E, editor. Pediatric Osteology: New Developments in Diagnostics and Therapy. Amsterdam: Elsevier; 1996:141-146.

187. Leggin BG, Neuman RM, Iannotti JP, Williams GR, Thompson EC. Intrarater and interrater reliability of three isometric dynamometers in assessing shoulder strength. J Shoulder Elbow Surg 1996;5(1):18-24.

188. Vandenborne K, Elliott MA, Walter GA et al. Longitudinal study of skeletal muscle adaptations during immobilization and rehabilitation. Muscle Nerve 1998;21(8):1006-1012.

189. Morris NM, Udry JR. Validation of a self-administered instrument to assess stage of adolescent development. J Youth Adolesc 1980;9:271-280.

190. Schall J, Semeao E, Stallings V, Zemel B. Validity of self-assessment of sexual maturity status in children and adolescents with Crohn's disease. J Pediatr 2002.

191. Zelikovsky N. Medication Adherence Measure. unpublished; 2001.

192. Varni JW, Seid M, Kurtin PS. PedsQL 4.0: reliability and validity of the Pediatric Quality of Life Inventory version 4.0 generic core scales in healthy and patient populations. Med Care 2001;39(8):800-812.

193. Modi AC, Quittner AL. Validation of a disease-specific measure of health-related quality of life for children with cystic fibrosis. J Pediatr Psychol 2003;28(8):535-545.

194. Zhang J, Quan H, Ng J, Stepanavage M. Some Statistical methods for Multiple Endpoints in Clinical Trials. Controlled Clinical Trials; 1997.

195. Zhang J, Quan H, Ng J, Stepanavage M. Some statistical methods for multiple endpoints in clinical trials. Controlled Clinical Trials 1997;18:204-221.

196. Laird N, Ware JH. Random-effects models for longitudinal data. Biometrics 1982;38:963-974.
