# Supplementary material for: Improved residual fat malabsorption and growth in children with cystic fibrosis treated with a novel oral structured lipid supplement: A randomized controlled trial
Source: PLoS One. 2020 May 8;15(5):e0232685. doi: 10.1371/journal.pone.0232685 (PMC7209323; doi:10.1371/journal.pone.0232685)
Supplement: Attachment — Submitted filename: PLOS LXS response to review 040920.docx [file pone.0232685.s004.docx]

PONE-D-19-35347R1
Improved Residual Fat Malabsorption and Growth in Children with Cystic Fibrosis Treated with a Novel Oral Structured Lipid Supplement: A Randomized Controlled Trial
PLOS ONE

Dear Dr. Tindall,

Thank you for submitting your manuscript to PLOS ONE. After careful consideration, we feel that it has merit but does not fully meet PLOS ONE’s publication criteria as it currently stands. Therefore, we invite you to submit a revised version of the manuscript that addresses the points raised during the review process.

We would appreciate receiving your revised manuscript by May 24 2020 11:59PM. When you are ready to submit your revision, log on to <https://www.editorialmanager.com/pone/> and select the 'Submissions Needing Revision' folder to locate your manuscript file.

If you would like to make changes to your financial disclosure, please include your updated statement in your cover letter.

To enhance the reproducibility of your results, we recommend that if applicable you deposit your laboratory protocols in protocols.io, where a protocol can be assigned its own identifier (DOI) such that it can be cited independently in the future. For instructions see: <http://journals.plos.org/plosone/s/submission-guidelines#loc-laboratory-protocols>

Please include the following items when submitting your revised manuscript:

- A rebuttal letter that responds to each point raised by the academic editor and reviewer(s). This letter should be uploaded as separate file and labeled 'Response to Reviewers'.
- A marked-up copy of your manuscript that highlights changes made to the original version. This file should be uploaded as separate file and labeled 'Revised Manuscript with Track Changes'.
- An unmarked version of your revised paper without tracked changes. This file should be uploaded as separate file and labeled 'Manuscript'.

Please note while forming your response, if your article is accepted, you may have the opportunity to make the peer review history publicly available. The record will include editor decision letters (with reviews) and your responses to reviewer comments. If eligible, we will contact you to opt in or out.

We look forward to receiving your revised manuscript.

Kind regards,

Maret G Traber, PhD
Academic Editor
PLOS ONE


Additional Editor Comments (if provided):

Please address the outstanding concerns from one of the reviewers, or provide a rationale why you think this action is unnecessary.


[Note: HTML markup is below. Please do not edit.]

Reviewers' comments:

Reviewer's Responses to Questions

**Comments to the Author**

1. If the authors have adequately addressed your comments raised in a previous round of review and you feel that this manuscript is now acceptable for publication, you may indicate that here to bypass the “Comments to the Author” section, enter your conflict of interest statement in the “Confidential to Editor” section, and submit your "Accept" recommendation.

Reviewer #2: (No Response)2. Is the manuscript technically sound, and do the data support the conclusions?

The manuscript must describe a technically sound piece of scientific research with data that supports the conclusions. Experiments must have been conducted rigorously, with appropriate controls, replication, and sample sizes. The conclusions must be drawn appropriately based on the data presented. 

Reviewer #2: Yes
3. Has the statistical analysis been performed appropriately and rigorously? 

Reviewer #2: Yes

4. Have the authors made all data underlying the findings in their manuscript fully available?

The [PLOS Data policy](http://www.plosone.org/static/policies.action#sharing) requires authors to make all data underlying the findings described in their manuscript fully available without restriction, with rare exception (please refer to the Data Availability Statement in the manuscript PDF file). The data should be provided as part of the manuscript or its supporting information, or deposited to a public repository. For example, in addition to summary statistics, the data points behind means, medians and variance measures should be available. If there are restrictions on publicly sharing data—e.g. participant privacy or use of data from a third party—those must be specified.

Reviewer #2: Yes
5. Is the manuscript presented in an intelligible fashion and written in standard English?

PLOS ONE does not copyedit accepted manuscripts, so the language in submitted articles must be clear, correct, and unambiguous. Any typographical or grammatical errors should be corrected at revision, so please note any specific errors here.

Reviewer #2: Yes6. Review Comments to the Author

Please use the space provided to explain your answers to the questions above. You may also include additional comments for the author, including concerns about dual publication, research ethics, or publication ethics. (Please upload your review as an attachment if it exceeds 20,000 characters)

**Reviewer #2:** The authors have addressed the concerns raised by this reviewer in their point-by-point reply and have added corresponding additions to their manuscript.

In contrast to the statement in the reply "As suggested, we added the total, saturated, monounsaturated and polyunsaturated dietary fat intake data to Table 2" and "There were no significant differences in intake between groups for total, monounsaturated fat, polyunsaturated fat, or saturated fat classes." these additional data do not appear in this table. These data should be added as described.

**We have amended the table to include these dietary fats and the statement "There were no significant differences in intake between groups for total, monounsaturated fat, polyunsaturated fat, or saturated fat classes” remains accurate.**

While it was proposed that "Regarding the unbalanced distribution of 63% of the treatment group showing CFA <88%, while only 39% did so in the placebo group ... this is related to the original (Encala/placebo group) randomization procedure for all subjects with sex/age blocks ... It did not include CFA, as CFA was an outcome variable ... We have included this in the limitations section", this explanation is still missing in the limitations section and should be appropriately addressed in a re-revised version of the manuscript.

**We have added the following sentence to the discussion: “The unbalanced distribution was related to the original Encala™/placebo randomization procedure for all subjects with sex/age blocks that did not include CFA, as CFA was an outcome variable.” (line 227-229 in the marked up version)**

Even though the inclusion/exclusion criteria have been previously reported (refs. 10-12), it would be helpful for the readers to include information regarding "Significant liver disease and any other significant diagnosis that might impact dietary intake, growth or body composition were exclusion criteria for subjects to participate in this study. There were no subjects with cholestatic liver disease" provided in the reply also in the manuscript as this is highly relevant for the present study.

**We have added the following sentence under “inclusion/exclusion criteria”: “Liver disease and any other significant diagnosis that might impact dietary intake, growth or body composition were exclusion criteria for subjects to participate in this study. There were no subjects with cholestatic liver disease.”**
